# Safety and Efficacy of the 10-Day Melarsoprol Schedule for the Treatment of Second Stage Rhodesiense Sleeping Sickness

**DOI:** 10.1371/journal.pntd.0001695

**Published:** 2012-08-28

**Authors:** Irene Kuepfer, Caecilia Schmid, Mpairwe Allan, Andrew Edielu, Emma P. Haary, Abbas Kakembo, Stafford Kibona, Johannes Blum, Christian Burri

**Affiliations:** 1 Swiss Tropical and Public Heath Institute, Basel, Switzerland; 2 University of Basel, Basel, Switzerland; 3 Lwala Hospital, Lwala, Uganda; 4 Kaliua Health Centre, Kaliua, Tanzaina; 5 Ministry of Health, Vector Control Division, Kampala, Uganda; 6 National Institute for Medical Research, Tabora Research Centre, Tabora, Tanzania; Foundation for Innovative New Diagnostics (FIND), Switzerland

## Abstract

**Objective:**

Assessment of the safety and efficacy of a 10-day melarsoprol schedule in second stage *T.b. rhodesiense* patients and the effect of suramin-pretreatment on the incidence of encephalopathic syndrome (ES) during melarsoprol therapy.

**Design:**

Sequential conduct of a proof-of-concept trial (n = 60) and a utilization study (n = 78) using historic controls as comparator.

**Setting:**

Two trial centres in the *T.b. rhodesiense* endemic regions of Tanzania and Uganda. Participants: Consenting patients with confirmed second stage disease and a minimum age of 6 years were eligible for participation. Unconscious and pregnant patients were excluded.

**Main Outcome Measures:**

The primary outcome measures were safety and efficacy at end of treatment. The secondary outcome measure was efficacy during follow-up after 3, 6 and 12 months.

**Results:**

The incidence of ES in the trial population was 11.2% (CI 5–17%) and 13% (CI 9–17%) in the historic data. The respective case fatality rates were 8.4% (CI 3–13.8%) and 9.3% (CI 6–12.6%). All patients discharged alive were free of parasites at end of treatment. Twelve months after discharge, 96% of patients were clinically cured. The mean hospitalization time was reduced from 29 to 13 days (p<0.0001) per patient.

**Conclusions:**

The 10-day melarsoprol schedule does not expose patients to a higher risk of ES or death than does treatment according to national schedules in current use. The efficacy of the 10-day melarsoprol schedule was highly satisfactory. No benefit could be attributed to the suramin pre-treatment.

**Trial Registration:**

Current Controlled Trials ISRCTN40537886

## Author Summary

Human African Trypanosomiasis (HAT) is a parasitic disease that occurs in a chronic form caused by *T.b. gambiense* in West and Central Africa and in an acute form caused by *T.b. rhodesiense* in East and South Africa. In the first stage of the disease parasites are detectable in blood and lymph. Once they enter the central nervous system, patients are in the so-called second stage, which is fatal if left untreated. Melarsoprol, an old and highly toxic drug, remains the only available treatment for second stage *T.b. rhodesiense* HAT. The empirically developed treatment regimens are a high burden to affected patients and health care providers due to very long hospitalization times and complicated dosing schemes. An improved 10-day melarsoprol treatment schedule was developed and tested in second stage *T.b. gambiense* patients from 1997–2004 and was approved for large-scale use by the International Scientific Committee for Trypanosomiasis Research and Control (ISCTRC) in 2003. To test its adequacy also in *T.b. rhodesiense* patients, a series of clinical trials were conducted from 2005–2009. Based on these results the ISCTRC has officially recommended the use of the 10-day melarsoprol schedule also for treatment of second stage Rhodesiense HAT in 2009.

## Introduction

Human African Trypanosomiasis (HAT), better known as sleeping sickness, is transmitted through tsetse flies *(Glossina ssp.)*. The disease occurs in a chronic form caused by *Trypanosoma brucei gambiense* (West and Central Africa) and an acute form caused by *Trypanosoma brucei rhodesiense* (East and South Africa). Both forms of HAT are fatal if left untreated. Time to death has been estimated at almost 3 years for *T.b. gambiense* [1] and at 6 to 12 months for *T.b. rhodesiense* infections [2].

Estimating the true prevalence of sleeping sickness is difficult as less than 10% of the at risk population are under adequate surveillance [3]. The majority of reported cases are *T.b. gambiense* infections [4], and *T.b. rhodesiense* HAT is clearly more neglected. However, rhodesiense sleeping sickness has a dangerous potential for large scale epidemics which are of high public health importance [5]. Between 1976 and 1998 a total of 19'974 cases were detected in south eastern Uganda [6], an area that today, has the potential for a much larger number of patients due to the expansion of HAT to previously disease free areas [7], [8]. The disease mainly affects local people but sporadic infections in tourists, especially in National Parks, occur and have spurred discussions about disease control and surveillance [9].

Sleeping sickness progresses from the first, haemolymphatic stage, where parasites are detectable in blood and lymph to the second, meningo-encephalitic stage where parasites enter the central nervous system. Clinical signs and symptoms of second stage disease are severe sleep disturbances, neurological and psychiatric disorders, coma, and death. A lumbar puncture is required for disease staging. The second stage is indicated by the presence of trypanosomes and/or elevated levels of white blood cells (≥5WBC/mm3) in the cerebrospinal fluid (CSF) [3]. For the treatment of *T.b. gambiense* HAT, pentamidine is used to treat the first stage and eflornithine, a combination of eflornithine and nifurtimox (NECT) or melarsoprol to treat second stage infections. *T.b. rhodesiense* infections are treated with suramin in the first, and melarsoprol in the second stage. Eflornithine can only be used for the treatment of *T.b. gambiense* HAT due to its limited activity against *T.b. rhodesiense* [10]. NECT has been recommended as new first line treatment for *T.b. gambiense* HAT [11] and was added to the WHO Essential Medicine List in 2009 [12]. The NECT implementation is currently ongoing in *T.b. gambiense* affected areas. For *T.b. rhodesiense* HAT, melarsoprol remains the only available drug to treat second stage infections.

Melarsoprol is a highly toxic, arsenical compound and was first introduced in 1949 [13]. Treatment regimens were empirically developed and vary considerably between countries and treatment centres. Complicated dosing schemes, based on serial drug applications separated by 1-week drug-free intervals, are common and result in hospitalization times of up to one month. The most severe complication of melarsoprol therapy is the encephalopathic syndrome (ES). It appears to be more common in *T.b. rhodesiense* than in *T.b. gambiense* HAT, with reported incidence rates ranging from 5–18% [14]. The concomitant use of steroids during melarsoprol therapy proved to reduce the incidence of ES in *T.b. gambiense* patients [15] but this could not be shown in *T.b. rhodesiense* [16], [17]. In East Africa, the heterogeneity of treatment protocols is further enhanced by the fact that some counties (Tanzania, Uganda, parts of Malawi) administer a suramin pre-treatment. It is given prior to the diagnostic lumbar puncture and should (i) prevent a mechanical introduction of trypanosomes into the CNS during the LP and (ii) clear trypanosomes from blood and lymph to avert initial high antigen releases at the initiation of melarsoprol therapy. Treatment protocols for the suramin pre-treatment are also heterogeneous and in practice, suramin is not given to critically ill patients to quickly reach curative melarsoprol concentrations in the CNS. The use of the suramin pre-treatment is purely empirical [18] and there is no solid evidence supporting this approach. Table 1 summarizes the national treatment schedules currently in use for the treatment of second stage *T.b. rhodesiense* HAT in East Africa. The total amount of melarsoprol administered and the hospitalization time are also shown.

**Table 1:** National treatment schedules for 2^nd^ stage *T.b. rhodesiense* HAT.

	Uganda	Tanzania	Kenya	Malawi
**Suramin pre-treatment (mg/kg)**				
1^st^ dose	5	5	NA	5
2^nd^ dose		20	NA	20
**Melarsoprol treatment (mg/kg)**				
1^st^ series	0.5, 0.72, 1.08	2.2, 2.52, 2.88	3.6, 3.6, 3.6	3.6, 3.6, 3.6
2^nd^ series	1.44, 2.80, 2.2	2.88, 3.24, 3.6	3.6, 3.6, 3.6	3.6, 3.6, 3.6
3^rd^ series	2.52, 2.88, 3.24	3.6, 3.6, 3.6	3.6, 3.6, 3.6	3.6, 3.6, 3.6
4^th^ series	3.6, 3.6, 3.6		3.6, 3.6, 3.6	
**Total melarsoprol (mg/kg)**	**27**	**28.08**	**43.2**	**32.4**
**Hospitalization time (days)**	**29**	**27**	**33**	**26**

NA:not applicable; i.v. melarsoprol injections at 24 hours intervals per series, each series spaced by 5 to 7 days resting periods; further variations of the schedules at local level.

The first, rational approach to a standardized melarsorpol treatment was the development of a treatment schedule based on pharmacokinetic investigations [19], [20]. The new schedule consists of daily melarsoprol injections (2.2 mg/kg) for 10 consecutive days. It was validated for second stage *T.b. gambiense* HAT in the framework of the Impamel I & II programs (1997–2004). Results showed that the abridged schedule is clinical non-inferior over the empirical standard regimens [21] and equally effective [22]. The 10-day schedule was clearly favored by patients and health personnel due to an >50% reduction in hospitalization time and a more economic use of the drug [23]. In 2003, the 10-day melarsoprol schedule was officially recommended for use in *T.b. gambiense* affected areas by the International Scientific Council for Trypanosomiasis Research and Control (ISCTRC).

Due to the significant differences in the two forms of HAT it was not possible to introduce the 10-day melarsoprol schedule in *T.b. rhodesiense* endemic areas without further testing. The WHO scientific working group recommended the urgent conduct of the necessary clinical trials in East Africa (2001), a call that was repeated by WHO Afro in 2003. The Impamel III program (2005–2009) was conducted as the first clinical trial program in accordance to international standards in *T.b. rhodesiense* patients. It assessed the safety and the efficacy of the 10-day melarsoprol schedule in second stage *T.b. rhodesiense* patients and the effect of suramin-pretreatment on incidence of serious adverse events during melarsoprol therapy. Given the limited number of *T.b. rhodesiense* patients, clinical trials are evidently restricted to small sample sizes and must be executed in rural settings with very limited infrastructure and difficult access. Randomized controlled trials or other trial designs with active control groups are therefore not feasible. The design of the Impamel III program was to conduct two sequential trials: first a proof-of-concept trial was conducted to proof no harm of the 10-day melarsoprol schedule in *T.b. rhodesiense* patients and to obtain preliminary efficacy data. Two subgroups, of which only one received suramin, allowed observing a possible, substantial increase of adverse drug reactions if melarsoprol was directly administered. Based on those findings, a second trial was designed to substantiate the results in a larger patient population. The second, drug utilization trial, was designed as an extension of the selected arm of the proof-of-concept trial, i.e. without suramin, thus, allowing pooling data from both trials for final analysis. Patient records from a maximum of two years prior to the Impamel III program were analyzed and used as comparator. The findings of the two sequentially conducted trials are reported here collectively.

## Methods

### Ethics Statement

Ethical clearance was obtained from the ethics committees in the host countries; the National Institute for Medical Research (NIMR), Tanzania and the Ministry of Health, Uganda. In Switzerland, ethical clearance was obtained from the ethics committee of the two cantons of Basel (EKBB). Each participant gave written informed consent. For the participation of children and adolescents (below 18 years) the parents, the legal representative or the guardian gave written informed consent. The trials were registered with Current Controlled Trials prior to first patient enrolment (ISRCTN40537886). The trials were conducted in compliance with ICH/GCP. For the use of historic controls, data was anonymized.

### Study Sites

The Kaliua Health Centre (KHC), a 50-bed missionary hospital in Tanzania (Urambo District) and the Lwala Hospital, a designated 100- bed district hospital in Uganda (Kaberamaido District) participated in the Impamel III program. Capacity building included on-site trainings in HAT diagnosis, Good Clinical Practice (GCP) and the upgrading of the laboratories and pharmacies.

### Study Design

Sequential conduct of two non-randomized trials; a proof-of-concept trial (n = 60) followed by a utilization study (n = 78) using historic data as comparator (n = 300).

#### Proof-of-concept trial (Protocol S1)

60 patients were prospectively enrolled into two subgroups: participants in the first subgroup (n = 30) were treated with the suramin pre-treatment followed by the 10-day melarsoprol schedule. The second sub group (n = 30) was directly treated with the 10-day melarsoprol schedule. Suramin and steroids were administered according to centre specific guidelines.

#### Utilization study (Protocol S2)

Additional 78 patients were treated with the 10-day melarsoprol schedule directly. The suramin pre-treatment was omitted and the use of steroids was adjusted to the Tanzanian standard in both centers (details below).

### Eligibility Criteria

Patients with confirmed second stage *T.b. rhodesiense* HAT and a minimum age of 6 years were eligible for participation. Pregnant as well as unconscious or moribund patients were excluded from the trial. Each participant gave written informed consent. For the participation of children and adolescents (below 18 years) the parents, the legal representative or the guardian gave written informed consent.

### Diagnosis and Staging

Diagnosis of HAT was made in blood and in the cerebrospinal fluid (CSF). Blood was examined using microscopy and/or the haematocrit centrifugation technique (WOO test) [24]. If trypanosomes were present, a lumbar puncture was performed for disease staging. Patients in the first subgroup of the proof-of-concept trial received the suramin pre-treatment prior to the LP. All other patients underwent LP directly after the detection of trypanosomes in blood. Analysis of the CSF was done by direct microscopy and/or single modified centrifugation technique and white blood cell (WBC) count using counting chambers. Second stage infections were confirmed by the presence of trypanosomes and/or ≥5 WBC/mm^3^ in the CSF.

### Patient Follow-Up

All patients were asked to present at the centre for follow-up examinations after 3, 6 and 12 months. At each follow-up visit blood and CSF samples were taken and were analyzed for the presence of trypanosomes. A WBC count was performed using the CSF sample. For patients who did not present for follow-up visits, oral information on their general condition was collected.

### Endpoints: Safety & Efficacy

Based on reported case fatality rates in the trial sites and the literature, a cut-off point of all-cause mortality was set at ≥10%. The computed safety stopping rule was an early discontinuation of the trials if 7 or more patients per subgroup (n = 30) experienced a fatal treatment outcome (p = 0.026).

The primary efficacy endpoint was cure at end of treatment. Secondary efficacy endpoint was cure after 3, 6 and 12 months. Possible outcome measures are summarized in Table 2.

**Table 2:** Possible safety and efficacy outcome measures of Impamel III trials.

Cure at end of treatment	No parasites in blood and CSF
Cure during follow-up	No parasites in blood and CSF AND WBC<5/mm^3^
Clinical cure at end of treatment	No parasites in blood but missing results on CSF analysis (refusal of LP, hemorrhagic LP)
Clinical cure during follow-up	No parasites in blood but missing results on CSF analysis (refusal of LP, hemorrhagic LP) OR Oral information on good general condition of the patient
Relapse (end of treatment and during follow-up)	Trypanosomes in any body fluid
Death	Patients who died during treatment or follow-up (categorized by likely or definite cause of death):
	- HAT
	- Adverse events regarded by the investigator as possibly or probably related to treatment for HAT
	- Causes unrelated to HAT or the treatment of HAT
	- Unknown causes

A high degree of homogeneity in the trial populations was achieved by the use of identical eligibility criteria, stopping rule and endpoints in both trials.

### Historic Controls

To reduce bias, historic controls were solely collected in the two trial sites and limited to a time frame of maximum two years prior to study initiation. Files that contained basic demographic data and information on treatment and treatment outcome were selected and checked for information on serious adverse events (SAEs), concomitant treatments and death. The same exclusion criteria as in the trial population were applied to historic controls. The files were sufficiently documented to exclude patients below 6 years of age and pregnant women. There was insufficient information to systematically exclude unconscious and moribund patients. A total of 300 files were used as historic controls (153 in Tanzania, 147 in Uganda).

### Sample size

No formal sample size was calculated for the proof-of-concept trial. The sample size calculation for the utilization was based on the accuracy of the occurrence of the safety endpoint. A minimum of 100 patients estimated the probability of the occurrence of all-cause mortality ≥10% with a standard error of 3%.

### Analysis Plan

In a first step both trials were analyzed separately. Final safety and efficacy analysis was performed on the pooled dataset of all patients directly treated with the 10-day melarsoprol schedule (n = 107). Those results were compared to historic controls.

### Recruitment

Patient recruitment was mainly by passive case detection at the centre. Active case search was done in villages of index cases, but the outcome was poor. In Uganda, the local radio station was contracted to inform the population about the Impamel III program and invited people to present for cost-free HAT screening at the Lwala hospital.

All trial participants were given an insecticide treated bed net (ITN). In case of need, the trial participants and/or their attendants were given food during the hospitalization period. Cost of transport for the patient and one attendant to present for follow-up visits was refunded.

### Data Management and Statistical Analysis

All data were double entered and verified using the EpiData Version 3.1 software (www.epidata.dk). Data analysis was done using the statistical software package STATA Version IC10.0 (STATA™, StataCorp, USA). Pearson's chi-square test and the Student's *t*-test were used to test differences in proportions and means.

### Trial Conduct

For each patient, a case report form (CRF) was filled containing information on demographic, diagnostic, and clinical characteristics before and after treatment. The assessment of adverse events used a graded scale for the severity of the event (0 to 4, absent to severe) and a binary outcome for the seriousness of the event. Signs and symptoms which were spontaneously reported between the end of treatment evaluation and 30 days post-treatment were also entered in the case report form.

During the proof-of-concept trial, the blood sugar and the blood lipids were monitored daily before food intake using the whole blood test system Cardio Chek™ PA. As suramin is nephrotoxic, proteinuria was monitored in all patients that received suramin at baseline and discharge examination as well as prior to the first melarsoprol application (using COMBUR9, Roche Diagnostics Switzerland).

During both trials, vital signs were daily monitored before drug administration. For women, a pregnancy test was performed at baseline.

Patients were treated with anti-malarial and anti-helminth drugs prior to HAT treatment in case the respective diagnosis was positive. During treatment, all patients received paracetamol (acetaminophen) 3 times per day in single doses of 1000 mg for adults and 500 mg or 250 mg respectively for children. Suramin was administered intravenously as a 10% aqueous solution (Germanin, Bayer). In the proof-of-concept trial, suramin as well as steroids were given according the centre-specific guidelines. In Tanzania, a suramin test dose (5 mg/kg) was administered after the detection of trypanosomes in blood (day 1). After a resting day, a full dose (20 mg/kg) was given on day 3. Another resting day followed before the LP on day 5. Each patient was treated with 10 mg of prednisone half an hour before each melarsoprol injection. In Uganda, patients received one suramin test dose (5 mg/kg) after the detection of trypanosomes in the blood. On the following day, the LP was performed and melarsoprol treatment was initiated. Steroids were only administered in case of reactions to treatment. Melarsoprol treatment was for all patients 2.2 mg/kg of melarsoprol for 10 consecutive days as a 3.6% solution in propylene glycol (Arsobal; Sanofi-aventis); by slow intravenous (i.v.) injection but maximally 5 ml a day. In the utilization study, all patients underwent LP directly after a trypanosome-positive blood test, the use of steroids was adapted to the Tanzanian standard and the use of suramin was omitted.

If a patient developed an encephalopathic syndrome (ES) defined by the occurrence of convulsions, and/or confusion, coma [25], melarsoprol treatment was interrupted and emergency treatment was initiated: i.v. hydrocortisone (100–200 mg/24 hours) or dexamethasone (3×15 mg/24 hours) and if necessary, anticonvulsive drugs (diazepam, phenobarbital) were administered. Close observation and frequent monitoring of vital signs were mandatory as well as supportive feeding, if necessary. For exclusion of cerebral malaria, blood was analyzed at the day of onset of the ES.

## Results

First, the results of the proof-of-concept trial are presented, followed by the results of the pooled data set and the comparison to the historic data.

**Figure 1:**
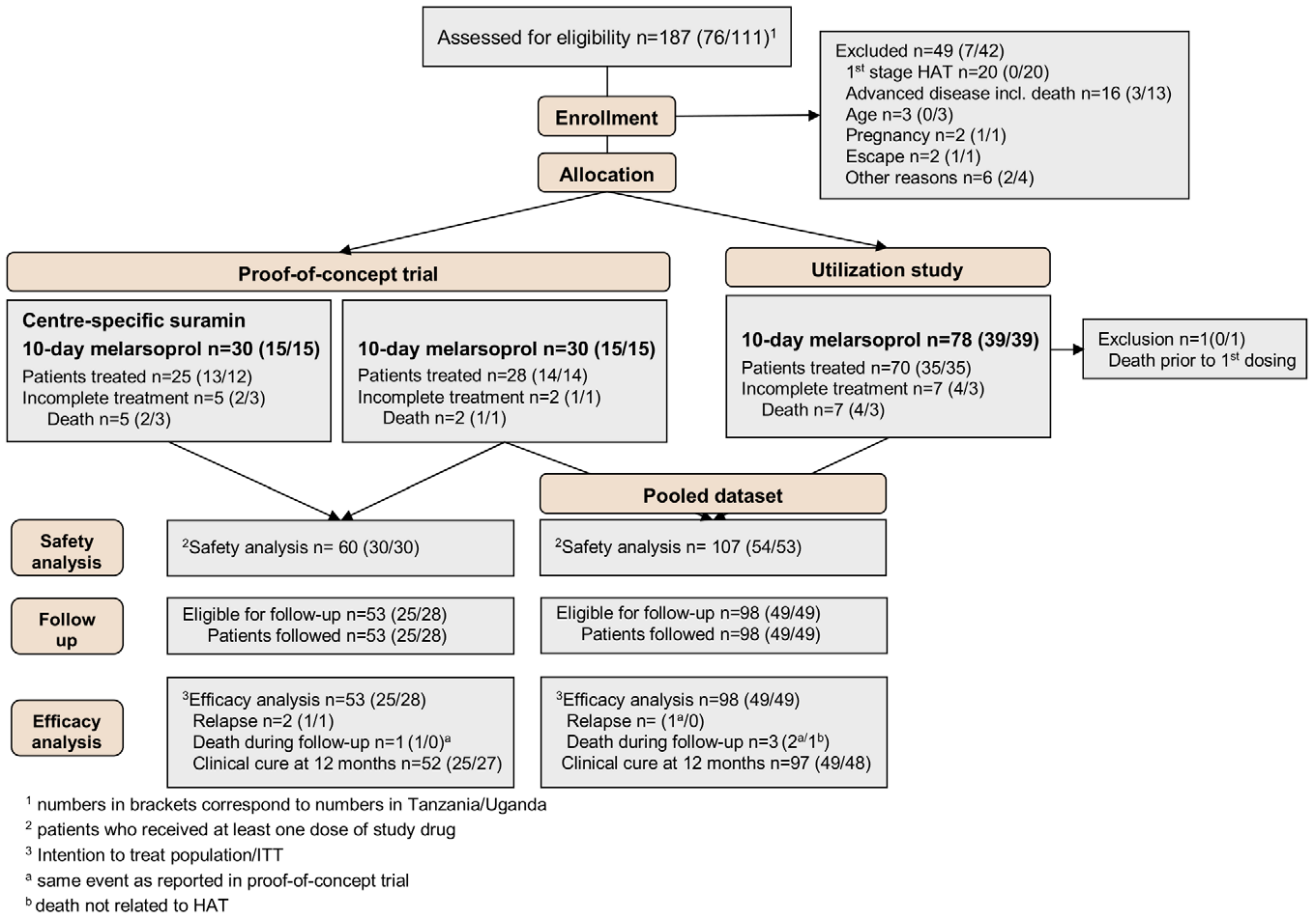
Overall study flow chart.

The study flow is presented in Figure 1.

### Proof-of-concept Trial

#### Study population and baseline characteristics

From August 2006 to July 2007 a total of 60 patients were enrolled. The age and sex distribution were similar in both sites. The median age in Uganda (31 years) was slightly lower than in Tanzania (36 years) as 10 participants were between 6 and 15 years of age. Malnutrition (BMI<16.5) was more common in Uganda (p = 0.001). In Tanzania, patients had less frequently trypanosomes in the CSF (p = 0.0035) but significant higher WBC counts (p<0.0001). The majority of patients suffered from headaches (90%), general malaise (93.3%) and joint pains (86.7%). Fever (axillary >37.5°C) at baseline was recorded in 35% of all patients.

#### Safety

A total of 13 serious adverse events (SAE) were reported whereof 7 (53.8%) were fatal. Other SAEs included prolonged hospitalizations and events that required medical interventions. More ES were reported in Uganda than in Tanzania (6 vs. 1; p = 0.0444). The time to onset of the ES was between 3 and 11 days after the first melarsoprol dose (median 6, mean 7). The overall survival rate for the ES was 28.6% (0% in Tanzania and 33% in Uganda). Adverse events reported included headache (15%), vomiting (13%), febrile reactions (13%), diarrhea (8.3%), nausea (6.6%), dizziness (5%), skin reactions (1.6%) and were controlled by symptomatic treatment. 56.7% of patients had an event free treatment course.

#### Efficacy

24 hours after treatment, all patients discharged alive (53/60) were free of parasites in blood and CSF. During follow-up, 2 relapses were reported, one from Tanzania and one from Uganda. The patient from Tanzania had been treated with melarsoprol only. 11 months after discharge he returned to the centre because he started to feel sick again after experiencing multiple tsetse bites and was diagnosed with second stage HAT. The patient from Uganda returned two weeks after discharge and was diagnosed with first stage HAT. He had been treated with suramin and melarsoprol and developed an ES after the 6^th^ injection of melarsoprol. The treatment was interrupted for 8 days and then resumed for the remaining 4 doses. Both relapse cases were successfully re-treated with melarsoprol according the national treatment schedules. Overall safety and efficacy outcomes of the proof-of-concept trial are summarized in Table 3. Results of blood sugar, blood lipids and urine analysis are not shown here.

**Table 3:** Proof-of-concept trial: safety & efficacy outcomes at discharge and follow-up.

	Total		Suramin		Non-suramin	
Patients treated	n = 60	%	n = 30	%	n = 30	%
Encephalopathic syndrome	7	11.6	4	13.3	3	10
Death during treatment	7	11.6	5^a^	16.6	2^a^	6.6
Relapses at discharge	0		0		0	
Cure at end of treatment	53	88.3	25	83.3	28	93.3

Patients eligible for follow-up	n = 53		n = 25		n = 28	
After 12 months						
Death	1	1.8	0		1^b^	3.6
Relapses	2	3.8	1	4.0	1	3.6
Clinical cure during follow-up	50	94.3	24	96	26	92.9

NOTE:

a two patients from Tanzania had an incomplete treatment and died outside the centre after family members took them back to the village to seek local treatment;

b not related to HAT (Tanzania).

Follow-up attendance was poorer in Tanzania, most probably due to longer distances to the health centre. 44% of the patients presented for the 3 months follow-up and 30% and 19% for the 6 and 12 months, respectively. In Uganda, 88% presented for the 3 months follow-up, 65% for the 6 months and 54% for the 12 months follow-up. For all patients not seen at the centre, oral information on their general condition was collected. All were in good condition and working, except one patient from Tanzania who died 7 months after discharge for reasons not related to HAT.

No benefit could be attributed to the suramin pre-treatment. In contrast, there were more ES and fatal treatment outcomes in the suramin group (see Table 3).

### Utilization Study

A total of 78 patients were enrolled from October 2007 to August 2008. For final analysis data from the proof-of-concept study (without suramin) and the utilization study were pooled. Table 4 compares the two patient populations prior to data pooling. None of the parameters were significantly different.

**Table 4:** Baseline characteristics of patient populations prior to data pooling.

	Proof-of-concept^1^		Utilization study		Pooled dataset	
	n = 30	%	N = 77	%	n = 107	%
Age, mean±SD	36±18		37±19		36±19	
Age, range (years)	6–67		6–72		6–72	
Male/female ratio	1.7		1.3		1.4	
Nutritional status						
BMI^2^ (kg/m) mean±SD	18.8±3.4		18.6±3.6		18.6±3.5	
Severe malnutrition (BMI<16.5)	8	26.6	18	23.4	26	24.3
Diagnostic findings						
Trypanosomes in blood	30	100.0	72	93.5	102	95.3
Trypanosomes in CSF^3^	28	93.33	69	89.6	97	90.7
WBC^4^ count in CSF	92±57		78±64		82±62	
Clinical manifestations						
Headache	27	90.0	73	94.8	100	93.5
Fever (>37.5)	7	23.3	13	16.9	20	18.7
Oedema	6	20.0	25	32.5	31	29.0
Joint pains	29	96.7	76	98.7	105	98.1
Daytime sleep	24	80.0	63	81.8	87	81.3
Night time sleep	23	76.7	50	64.9	73	68.2
Abnormal movements	8	26.7	20	26.0	28	26.2
Walking difficulties	13	43.3	53	68.8	66	61.7
Time period of enrolment	Oct 06–May 07		Oct 07–Aug 08		Oct 06–Aug 08	

Note:

1 no suramin pre-treatment;

2 body mass index,

3 cerebrospinal fluid,

4 white blood cell.

#### Study population and baseline characteristics

The demographic, diagnostic, and clinical characteristics of the patients were similar in Tanzania (Tz) and Uganda (Ug). 19 trial participants (Tz: 2, Ug: 17) were below 16 years of age with a mean age of 11 years (±3 years). Malnutrition (BMI<16.5) was significantly more frequent in Uganda (p<0.0001). In Tanzania, patients had less frequently trypanosomes in the CSF (p = 0.0002) but significantly higher WBC counts (p<0.0001). In line with the significant higher WBC count, the neurological symptoms were more distinct in Tanzanian patients. The clinical presentation of the disease in the two geographically distinct areas has been published separately [26].

#### Safety

**Figure 2:**
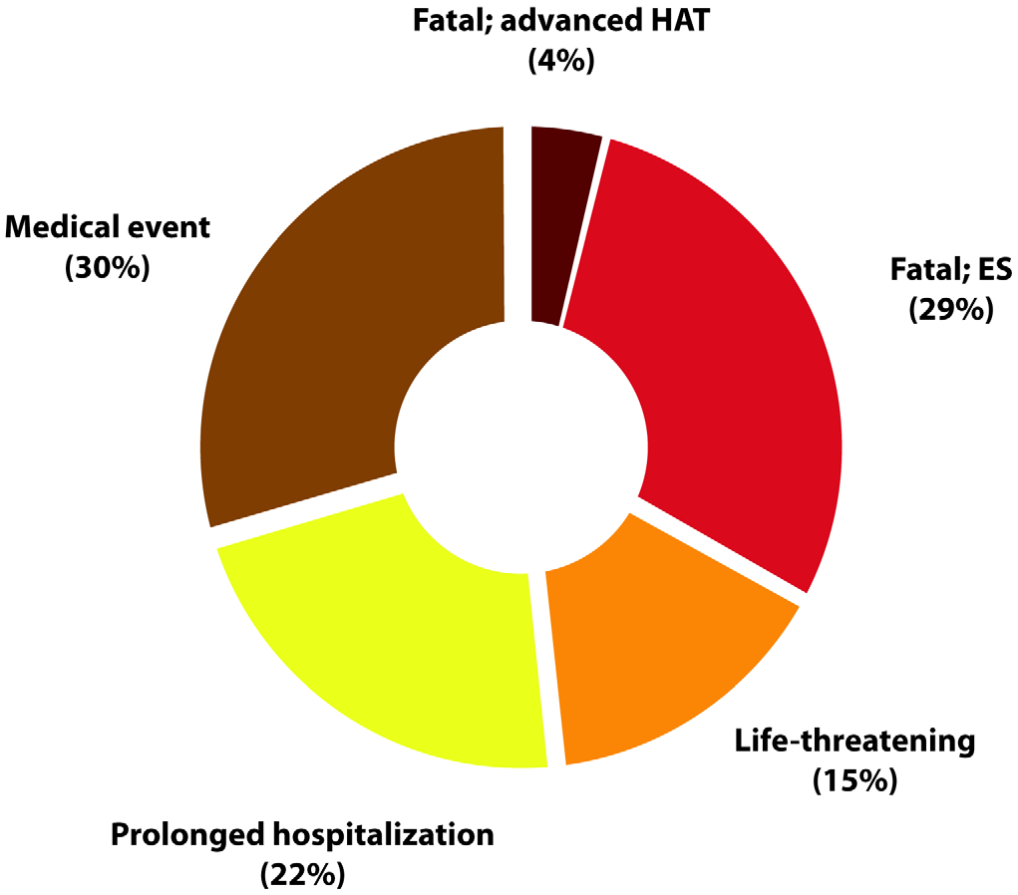
Serious adverse events, by SAE criterion.

A total of 27 SAEs were reported, summarized by SAE criterion in Figure 2. 33.3% (9/27) of the SAEs were fatal and included one death due to advanced HAT and 8 deaths due to ES. 14.8% (4/27) SAEs were life threatening events (non-fatal ES). 22.2% (6/27) SAEs were based on prolonged hospitalizations of patients who were kept for observation due to general weakness. 8 29.6% (8/27) SAEs were medical events and included treatment of malaria, severe vomiting, severe headache, cardiac arrhythmia and psychosis at end of treatment.

9 patients died during treatment. Death occurred between 2 and 16 days (median 9, mean 8.5) after the first injection of melarsoprol and the major cause was ES (88.9%). The onset of ES was reported after an average of 7.5 days after the first dose of melarsoprol (range 3–10 days). The onset was sudden, in 58.3% (7/12) preceded by headache and fever and in 41.6% (5/12) by vomiting. In 16.6% (2/12) malaria parasites were detected at the onset of ES, which probably also caused fever and headache. Differences were observed in the duration of ES; in Tanzania they were fatal after a maximum of one day and in Uganda the ES could last for several days (range 1–8) until the patient's condition improved or deteriorated. The overall ES survival rate was 33.3% (Tz: 0%; Ug: 57.1%).

Other adverse events reported included febrile reactions (37%), headache (22%), vomiting (13%), dizziness (9%), skin reactions (6.5%), nausea (5.6%) and diarrhea (4%). 35.5% the patients had an event-free treatment.

#### Efficacy

All patients discharged alive (98/107) were free of parasites in blood and CSF 24 hours after treatment. The follow-up attendance was better in the utilization study, most likely due to a better understanding of its importance. Follow-up attendance rates in Tanzania were 69% at the 3 months, 97% at the 6 months and 34% at the 12 months follow up. In Uganda 91% presented for the 3 months follow-up, 46% for the 6 months and 57% for the 12 months follow-up. For all patients that did not present at the centre, oral information on their well being was collected.

During follow-up, two deaths occurred in Tanzania whereof one occurred in a patient enrolled into the proof-of-concept trial (see above). The patient from Tanzania enrolled into the utilization study died 9 months after discharge due to reasons not related to HAT. One patient from Uganda died 2 months after discharge of unknown reasons. Table 5 summarizes the main safety and efficacy outcomes of the pooled data set at discharge and at 12 months after treatment.

**Table 5:** Utilization trial: safety & efficacy outcomes at discharge and follow-up.

	Total		Tanzania		Uganda	
Number of patients treated	n = 107	%	n = 54	%	n = 53	%
Encephalopathic syndrome	12	11.2	5	9.3	7	13.2
Death during treatment	9	8.4	5	9.3	4	7.5
Relapses at discharge	0		0		0	
Clinical cure at discharge	98	91.6	49	90.7	49	92.5
Patients eligible for follow-up	98		49		49	
At 12 months						
Death	3	3.1	2^a^	4.1	1	2
Relapses	1	1.0	1^a^	2.0	0	
Clinical cure	94	95.9	46	93.9	48	98.0

NOTE:

a one death and relapse enrolled and reported in proof-of-concept trial.

### Historic Data and Comparison to Trial Data

In both centers all HAT patient files from 2004–2006 were reviewed. A total of 300 files were selected, excluded were incomplete patient files (missing information on demographics and/or treatment evolution). The demographics, the incidence of ES and death as reported in the historic patient files are shown in Table 6.

**Table 6:** Demographics and incidence of ES and death in historic patient files.

	Total		Tanzania		Uganda	
Number of patients	n = 300	%	n = 153	%	N = 147	%
Age, mean ± SD	29±16		34±17^1^		25±17^2^	
Male/female ratio	1.4		2.5		0.8	
Encephalopathic syndrome	39	13.0	17	11.1	22	15.0
Death	28	9.3	12	7.8	16	10.9

1 missing values for age: 18,

2 missing values for age: 3.

In the historic data the mean reported incidence of ES in the historic data was 13% (Tz: 11.1%, Ug: 15%), of which 67.9% were fatal. The hospitalization time was 27 days in Tanzania and 32 days in Uganda (range 3–92). For trial patients directly treated with melarsoprol, the mean hospitalization time was 13 days (range 3–34) (p<0.0001). Given the significantly shorter hospitalization time, fewer patients in the trial population left the hospital prior to completion of treatment than compared to historic control (99% vs. 97%).

**Figure 3:**
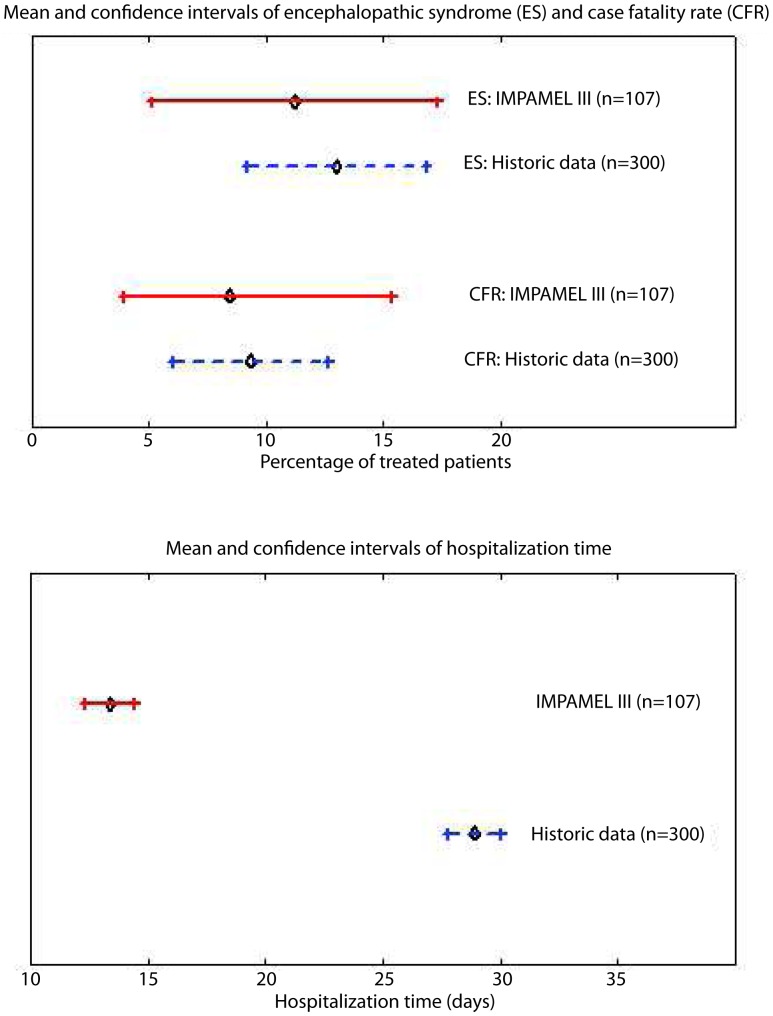
Mean and 95% CI for ES, CFR and hospitalization time. Trial data (solid line) and historic data (dashed line).

Comparison between trial and historic data for ES, case fatality rate (CFR) and hospitalization time is shown in Figure 3.

## Discussion

Given the significant differences between the chronic and acute form of HAT the results of the Impamel I&II programs could not be directly extrapolated to *T.b. rhodesiense* HAT and further testing was needed. In the planning of the Impamel III program, treatment efficacy was not the major concern as the total exposure time of the parasite to melarsoprol is similar in the empirical schedules and the Impamel schedule: in Tanzania, melarsoprol is given for 9 days (3×3) and in Uganda for 12 days (3×4), spaced by resting periods, versus a total of 10 consecutive days according the abridged schedule. Also, the Impamel schedule was extensively tested in second stage *T.b. gambiense* patients and yielded similar cure rates as the empirical schedules [22]. The main concerns were related to unexpected toxicity: given the already higher parasitaemia and reported incidence of ES in *T.b. rhodesiense* patients, a further increase of ES under the 10-day melarsoprol schedule could not be excluded. However, evidence from studies in *T.b. gambiense* HAT showed that the pathogenesis of ES is an immune phenomenon and dose independent [27]. Hence, the 10-day melarsoprol schedule should theoretically not trigger an increase in the incidence of ES in *T.b. rhodesiense* patients.

### Suramin Pre-treatment

Pre-treatment with pentamidine and suramin have been given for decades in the hope of reducing the risk for ES, but this remains unproven [14]. In Tanzania, and the southern parts of Malawi the suramin pre-treatment consist of a test dose (5 mg/kg) and a full dose (20 mg/kg) administered over a time period of 5 days. In Uganda, a test dose (5 mg/kg) is given the day before the LP which, from a pharmacological point of view, is unlikely to efficiently clear trypanosomes as suramin is only taken up slowly by the parasite [28]. The suramin pre-treatment is not given to critically ill patients to quickly reach antitrypanosomal activity in the CNS. In Kenya and northern parts of Malawi the suramin pre-treatment is not part of second stage HAT treatment protocols.

Because it was not possible to deviate from current national treatment protocols in one step, the assessment of the ability of the suramin pre-treatment to prevent adverse drug reactions was part of the Impamel III trial design, despite the lack of scientific evidence. We observed more adverse events during the proof-of-concept trial in patients that received suramin (63.3%) than in patients that were directly treated with melarsoprol (23.3%, p = 0.0018). Based on this result we decided to omit the suramin pre-treatment in the utilization study. In the pooled dataset, where none of the patients received suramin, 61.7% of the patients experienced adverse events. We observed no benefit of suramin pre-treatment over the direct melarsoprol application.

### Safety

35% of all patients treated directly with melarsoprol had an event-free treatment course. Concomitant treatments were less frequently used in the trial population compared to the historic data (p = 0.0001). Adverse events such as vomiting, headache, skin reactions and fever were controllable with concomitant medications. Skin reactions (rashes, pruritus) were a minor problem, reported in 6.5% of the patients. This was surprisingly low given the high incidence of severe skin reactions in *T.b. gambiense* patients (28.4%) of which 4.2% were fatal (bullous eruptions) [23]. The most relevant safety outcome of melarsoprol treatments is the incidence of serious adverse events (ES and death). A systematic literature review on encephalopathic syndromes during melarsoprol treatment of HAT [29] reported incidence rates of ES and death in *T.b. rhodesiense* patients of 10.6% (1.5–28%) and 11.6% (CI 5.2–19%) respectively. This compared well to the historic data where we report incidence rates of ES and death of 13% (CI 9.2–16.8%) and 9.3% (CI 6.0–12.6). In the trial population the incidence rates of ES and death were 11.2% (CI 5.1–17.3) and 8.4% (CI 3–13.8), respectively.

Findings from *T.b. gambiense* HAT of a higher risk for ES associated with the presence of trypanosomes or more than 100 WBC in CSF [15] could not be confirmed. However, the ES in Tanzania seemed more severe. Despite same standards of patient care and management the case fatality rate of ES was higher in Tanzania. Further investigations are needed to explain these differences. An immunological background of ES was suspected for long and recent investigations indicating that a small number of alleles of the human leukocyte antigen (HLA) were associated with a significantly increased risk for ES have corroborated this hypothesis [29]. During the proof-of-concept trial, patients from Uganda developed more ES than patients in Tanzania (p = 0.0444). However, the incidence of ES equilibrated between the centers in the utilization study (p = 0.5176) when steroids were administered in both centers according the same guidelines. The evidence for the prevention of ES with steroids in the literature is conflicting [16], [27], however, our data suggest a correlation between the frequency of ES and the use of prednisolone. However, the Impamel III program used a low dose of prednisolone (10 mg). In order to optimize its benefit in the prevention of ES the use or evaluation of a higher dose is recommended.

Causes of death are difficult to establish under field conditions. To avoid bias we used a composite safety endpoint of all-cause mortality, which is also better suited for comparison of mortality rates from literature and the historic data. We considered the historic data from a maximum time period of two years prior to the trial conduct as the most adequate source for controls, even though the quality and reporting standards were poorer than the comprehensive trial documentation.

### Efficacy

The historic data did not allow any elucidation of the efficacy of the standard treatment regimens at end of treatment and/or during follow up. Blood and CSF examinations at discharge and a systematic follow-up of patients are not routinely done. Due to the need for repeated LPs and the long distances to the health centers the follow-up attendance is generally very low. In *T.b. gambiense* endemic regions, patient follow-up is successfully supported by mobile teams that routinely do large-scale population screenings. Such teams are inexistent in *T.b. rhodesiense* areas and other approaches are needed to support patient follow-up. We engaged local leaders, community health workers and priests to collect information on the well-being of patients that did not present for follow-up examinations. However, to anticipate missing data, the primary efficacy endpoint was parasitological cure at end of treatment and the secondary efficacy endpoint was parasitological cure at follow-up examinations. Given the acuteness of this disease, relapses are certainly noted by the patients and communities but not necessarily reported. Therefore, oral information on the well being of the patients was very valuable and a satisfactory tool to determine treatment efficacy in the absence of blood and CSF examinations.

All patients discharge alive (121/137) were free of parasites in blood and CSF 24 hours after treatment. 12 months after discharge, the clinical cure rate was 94% in patients enrolled into the proof-of-concept trial and 96% in all patients directly treated with melarsoprol. 3 patients died during follow-up (2 in Tanzania, one in Uganda). 2 deaths were not related to HAT and one had an unknown cause of death. Overall, 2 relapses were reported, one in a patient treated with suramin and melarsorpol and one in a patient directly treated with melarsorpol. In one case a re-infection was most probable and in the other case, melarsorpol treatment had to be interrupted for 8 days, before the remaining 4 doses were administered. Further investigations are needed to identify the duration of treatment interruption which will require re-treatment with a full course. Overall, we report a relapse rate of 0.9% (1/107) in patients directly treated with melarsoprol. The relapse rate for the 10-day melarsoprol schedule in *T.b. gambiense* HAT was reported at 7.1% in a controlled clinical trial [23].

The Impamel III program was the first clinical trial program in *T.b rhodesiense* HAT conducted in compliance with international standards (ICH-GCP) and was of high priority by the WHO and the affected countries. The conduct of the trials strengthened local capacities, especially for diagnosis, patient management and reporting. The main bottleneck of clinical research in *T.b. rhodesiense* HAT is the overall limited number of patients. A sample size of minimum 400 patients (200 per arm) would have been required for the conduct of the Impamel III program in the design of a randomized control trial. But in two active foci, a total of 138 second stage patients were identified during two years of active and passive case detection, actively engaging with communities, district officials for vector control and disease surveillance. Poor accessibility of affected populations to health care facilities, the lack of health staff detecting HAT and low sensitivity of the diagnostic tools hamper case detection. Many patients die undetected, it is estimated that for each reported death, 12 go undetected [6].

Today, the biggest need for HAT affected populations is a new and safe treatment alternative. This will sadly not be the case in the near future and melarsoprol will continue to play the central role for the treatment of *T.b. rhodesiense HAT*. Given the increasing rates of melarsoprol treatment failures reported in *T.b. gambiense* patients [30]–[33], it can not be excluded that melarsoprol will also loose some of its efficacy against *T.b.rhodesiense*. Melarsoprol treatment failures have been reported in *T.b. rhodesiense* patients [34]–[37] as well as a reduced melarsoprol susceptibility of *T.b. rhodesiense* isolates from Tanzania, indicating that drug resistance may be emerging [38]. Besides the development of novel compounds, potential combination treatments for second stage *T.b. rhodesiense* HAT should be a high priority on the research agenda.

The results from the Impamel III program show that *T.b. rhodesiense* patients treated with the 10-day melarsoprol schedule were not subject to a higher incidence of serious adverse events (ES or death) than the historic controls treated with the national regimens (see Figure 3). In terms of efficacy, we have no evidence against the use of the 10-day melarsoprol schedule for the treatment of second stage *T.b. rhodesiense* HAT. The final data set used as historic controls was of very good quality. The only limitation was that the historic controls might have included more severely sick patients as the patient files did not in detail state if the patient was unconscious or moribund. The hospitalization time was reduced from an average of 29 days to 13 days (p<0.0001) and, together with the fixed dosing of 2.2 mg/kg/day, improves adherence to treatment from the patient and the provider side; fewer patients abandon treatment due to the financial burden of a one month hospital stay and at provider level, significantly less dosing mistakes are made compared to the varying dosing schemes of the national regimens. Our data further support the omission of the suramin-pre-treatment and a standardized use of steroids.

The 10-day schedule offers substantial advantages to the patients and the health care provider, is a basis for potential combination treatments and allows harmonizing the highly heterogeneous treatment protocols currently in use. However, as in *T.b. gambiense* HAT, ES still occur and continue to pose a major threat to the patients treated. Based on the Impamel III results the 10-day schedule was officially recommended for use in all *T.b. rhodesiense* endemic areas at the 30^th^ ISCTRC meeting in Kampala [39] and respective policy changes are currently ongoing in Tanzania and Uganda.

## Supporting Information

Protocol S1 **Study protocol proof-of-concept trial.**
(PDF)

Protocol S2 **Study protocol utilization study.**
(PDF)
