# Supplementary material for: Safety and Efficacy of the 10-Day Melarsoprol Schedule for the Treatment of Second Stage Rhodesiense Sleeping Sickness
Source: PLoS Negl Trop Dis. 2012 Aug 28;6(8):e1695. doi: 10.1371/journal.pntd.0001695 (PMC3435133; doi:10.1371/journal.pntd.0001695)
Supplement: Protocol S2 — Study protocol utilization study. (PDF) [file pntd.0001695.s002.pdf]

IMPAMEL III

Assessment of an abridged Melarsoprol Treatment Schedule against late stage *T.b. rhodesiense* Sleeping Sickness

Multinational Utilization Study

STUDY PROTOCOL

Incorporating Amendment#2 (26.07.2007)

Protocol Number:	P-001-05-01-03
Document date:	26.07.2007
Study Director:	PD Dr. Christian Burri<br>Swiss Tropical Institute<br>Socinstrasse 57<br>CH-4002 Basel, Switzerland<br>Tel +41 61 225 26 61<br>Fax +41 61 225 26 78<br>E-mail: Christian.Burri@unibas.ch
Study coordinator:	Irene Kuepfer<br>Swiss Tropical Institute<br>Socinstrasse 57<br>CH-4002 Basel, Switzerland<br>Tel +41 61 225 26 68<br>Fax +41 61 225 26 78<br>E-mail: Irene.Kuepfer@unibas.ch
Sponsor contact:	Swiss Tropical Institute<br>Socinstrasse 57,<br>CH-4002 Basel, Switzerland

CONFIDENTIALITY STATEMENT

This document contains information which is confidential and therefore is provided to you in confidence for review by you, your staff, an applicable Ethics Committee and regulatory authorities. It is understood that this information will not be disclosed to others without prior written approval from the Swiss Tropical Institute, except to the extent necessary to obtain informed consent from those persons to whom the drug may be administered.

1.1. Signatures of Agreement for Protocol

Christian Burri, MSc, PhD, Study Director, Head of CRO Date

Swiss Tropical Institute, CH-4002 Basel, Switzerland

J. Blum, MD, Principal Investigator Date

Swiss Tropical Institute, CH-4002 Basel, Switzerland

PROTOCOL AMENDMENT FOR:

P-001-05-01-02 – STUDY PROTOCOL FOR A MULTINATIONAL PHASE II STUDY (PROOF-OF-CONCEPT) OF THE ASSESSMENT OF AN ABRIDGED MELARSOPROL SCHEDULE AGAINST LATE STAGE *T. B. RHODESIENSE* SLEEPING SICKNESS.

Abstract

Sleeping sickness caused by *Trypanosoma brucei rhodesiense (T.b. rhodesiense)* has a very fast and acute progression, being fatal in order of weeks to a few months when left untreated. Melarsoprol remains the only drug for the treatment of the late stage of the disease. Melarsoprol is very toxic and has to be administered parenterally on a repeated basis. Serious adverse drug reactions and long treatment schedules result in high case fatality rates, low treatment quality and compliance. Between 1996 and 2004 the safety, efficacy and effectiveness of a new, abridged melarsoprol treatment has been investigated in countries affected by *Trypanosoma brucei gambiense (T.b. gambiense).* Due to its considerable socio-economic benefits, better cost-effectiveness and clinical non-inferiority, the 10-day melarsoprol treatment schedule was adopted as new, national treatment policy in countries fighting *T.b. gambiense* infections at the International Scientific Committee for Trypanosomiasis Research and Control / ISCTRC meeting in 2003.

Between August 2006 and June 2007 the IMPAMEL III proof-of-concept trial has been conducted in Tanzania and Uganda. A total of 60 late stage *T. b. rhodesiense* patients have been treated with the 10-day melarsoprol schedule. 50% of those patients (n=30) have received the centre-specific suramin pre-treatment prior to the first dose of melarsoprol. Preliminary results have shown that (i) the suramin pre-treatment has no apparent positive impact on the treatment of late stage *T. b. rhodesiense* sleeping sickness and (ii) there are no atypical issues concerning the safety, tolerability and efficacy of the 10-day schedule. However, more data is needed to further assess the safety, tolerability and efficacy of the 10-day melarsoprol schedule without suramin pre-treatment. However, more data is needed and the conduct of a multinational utilization study using historic patient data as a surrogate comparator represents the most appropriate and feasible approach.

Design

This is a two-centre, multi-national utilization study. A minimum of 70 subjects (≥6 years of age) are enrolled in 2 trial sites (Kaliua Health Centre, Tanzania and Lwala Hospital, Uganda) in order to evaluate the primary efficacy endpoint (end of treatment) of the abridged 10-day melarsoprol treatment without suramin pre-treatment against late stage T.b. rhodesiense sleeping sickness.

Objective

The objective of this study is to further assess the safety, tolerability and efficacy of the abridged 10-day melarsoprol treatment schedule without suramin pre-treatment in late stage *T.b. rhodesiense* patients.

2. Table of Contents

1.1. Signatures of Agreement for Protocol 1
Abstract 2
2. Table of Contents 3
3. Introduction 7
3.1. Overview of Human African Trypanosomiasis 7
3.2. Treatment of HAT 9
3.2.1. General Aspects 9
3.2.2. Melarsoprol Treatment 9
3.2.3. Development of a new, abridged melarsoprol schedule against late stage infections 11
3.2.3.1. Safety, efficacy and effectiveness of the IMPAMEL schedule 11
3.3. HAT Situation in East Africa 12
3.3.1. Uganda 13
3.3.2. Tanzania 13
3.3.3. Malawi 14
3.3.4. Situation in other East African countries 14
3.3.5. Current Treatment Protocols in use in Eastern Africa 15
3.4. Conclusion and Justification 16
4. Objectives 19
4.1. Primary Objective 19
4.2. Secondary Objective .... Error! Bookmark not defined.
4.3. Third Objective 19
5. Investigational Plan 19
5.1. Overall Study Design 19
5.1.1. Efficacy 20
5.1.2. Safety 21
5.1.3. Sub-Study 21
5.1.3.1. Blood sample collection for PCR-analysis 21
5.1.4. Discussion of Study Design 21
5.2. Study Population 23
5.2.1. Criteria for Inclusion 23
5.2.2. Criteria for Exclusion 23
5.2.3. HIV 24
5.2.4. Criteria for Discontinuation 24
5.2.4.1. Discontinuation of Individual Subjects 24

5.2.4.2. Discontinuation of the Entire Study ... 24
5.2.4.3. Scientific Advisory Board ... 24
5.3. Treatment ... 25
5.3.1. Treatment schedule ... 25
5.3.2. Additional Medication ... 26
5.3.3. Drugs to avoid during the study ... 26
5.3.4. Supplementary medication before melarsoprol treatment (standard if no rules exist) ... 26
5.3.5. Supplementary medication during melarsoprol treatment ... 26
5.4. Procedure in case of severe adverse events ... 26
5.4.1. Encephalopathy ... 26
5.4.2. Other adverse events ... 27
5.4.2.1. Neuropathies ... 27
5.4.2.2. Skin Eruptions ... 27
5.4.2.3. Hypotension or collapse ... 27
5.5. Compliance ... 28
6. Study Plan ... 28
6.1.1. Pre-treatment Period ... 28
6.1.2. Treatment Period ... 30
6.1.2.1. Study Drug administration ... 30
6.1.2.2. Procedures and Observations ... 30
6.1.3. End of Treatment ... 30
6.1.3.1. Subject Long-Term Follow-up ... 31
6.1.4. Monitoring for Safety ... 32
6.1.5. Collection and Reporting of Adverse Events ... 33
6.2. Efficacy and Safety Variables ... 34
6.2.1. Appropriateness of Measurements ... 34
6.2.2. Primary Efficacy Variables ... 34
6.2.2.1. Secondary Efficacy Variables ... 34
6.2.3. Safety Variables ... 35
7. Statistical Methods ... 36
7.1. Justification of Sample Size ... 36
7.2. Efficacy Analysis ... 36
7.3. Safety and Tolerability Analysis ... 36
7.4. Stopping rule ... 36
7.5. Statistical and Analytical Plan ... 36
8. Protocol Deviations ... 36

9. Ethics and Regulatory Requirements 37
9.1. Ethical Clearance 37
9.2. Ethical Conduct of the Study 37
9.3. Subject Information and Consent 38
9.4. Subject Confidentiality 38
10. Other Administrative and Regulatory Procedures 38
10.1. Data Quality Assurance 38
10.2. Source Documents, Case Report Forms (CRFs) 38
10.3. Maintenance of Records 39
10.4. Completion of the Study 39
10.4.1. Final Report 40
10.4.2. Use of Information 40
10.5. Publication 40
10.6. Country Specific Investigator’s Signature Page 41
Appendix 1 Glasgow Coma Scale (adapted) 42
Appendix 2 Grading of Clinical Signs and Symptoms of HAT 43
Appendix 3 Toxicity Grading Scale 44
Appendix 4 Administrative Procedures for the Reporting of Adverse Events 47
Appendix 5 Ethical Consideration and Human Subject Protection 50
Appendix 6 Essential Documents 54
11. References 55

Abbreviations used

BBB	Blood Brain Barrier
CNS	Central Nervous System
CRF	Case Report Form
CSF	Cerebrospinal fluid
ES	Encephalopathic syndrome
HLA	Human Leukocyte Antigen System
ISCTRC	International Scientific Committee for Trypanosomiasis Research and Control
IDP's	Internally Displaced People
i.v.	Intravenous
LIRI	Livestock Health Research Institute, Uganda
LP	Lumbar puncture
HAT	Human African Trypanosomiasis
HGB	Haemoglobin
MoH	Ministry of Health
NIMR	National Institute for Medical Research (Tanzania)
PCR	Polymerase Chain Reaction
p.o.	per os
SAB	Scientific Advisory Board
SRA-gene	Serum-resistance-associated gene
SS	Sleeping Sickness
STI	Swiss Tropical Institute
WBC	White blood cells (leucocytes)
WHO	World Health Organization
WNL	Within normal limits

3. Introduction

3.1. Overview of Human African Trypanosomiasis

Human African Trypanosomiasis (HAT), better known as sleeping sickness is caused by the protozoan parasites *Trypanosoma brucei gambiense* (West African form of the disease) and *Trypanosoma brucei rhodesiense* (East African form of the disease). The occurrence of the two species is more or less separated by the Great Rift Valley. *T.b. gambiense* is a very homogeneous parasite species whereas *T.b. rhodesiense* is very heterogeneous. *T.b. rhodesiense* is a zoonotic disease; host animals are game animals as well as domestic livestock. Both parasites are transmitted in the saliva of blood-sucking female tsetse flies (*Glossina spp.*), found in a belt that stretches between South of the Sahara and North of the Kalahari. During the 1950's, sleeping sickness was practically brought under control. Following the attainment of independence from colonial rule, sleeping sickness control has not been given sufficient attention by national health authorities. Social, political and economical development of rural areas, increased population movements, civil disturbances and reduced willingness of the people to submit to medical examination, yet aggravated this situation. The prevalence rate of trypanosomiasis rose again from 0.01 % of the population up to 1-2 %[1]. Currently, the disease affects 36 out of 53 African countries where all, or part of the population, is at risk of infection. Some 60 million people live at risk of contracting the disease, but no more than 3 to 4 million are under adequate surveillance[2]. WHO estimates that at least 300’000 individuals are infected, and the annual incidence of reported new cases is approximately 20’000 – 40’000 which certainly is an underestimation[2]. However, the importance of HAT with regard to public health lies not in the annual incidence, but in its potential for development of explosive epidemics[3]. If only the incidence is considered, HAT appears to be a minor health problem compared to other parasitic diseases like malaria and helminthic infections. However, even single cases or endemic outbreaks can severely affect whole families, the local economic situation and large scale epidemics were fatal for many thousand people in the past. If the DALY figures (e.g. loss of healthy life years by premature mortality and disability) are considered, the social and economic impact of trypanosomiasis even ranks third of all parasitic diseases behind malaria and schistosomiasis in sub-Saharan Africa[4].

The clinical profile of HAT is too diffuse to allow for a direct differential diagnosis. Only the proof of the parasites presence in blood, lymph nodes or cerebrospinal fluid is sufficient. *T.b. gambiense* can be sero-diagnosed with the CATT test (Card Agglutination Test) followed by microscopic confirmation of the parasite in blood and/or lymph. The diagnosis of *T.b. rhodesiense* has to entirely rely on microscopy. The sensitivity of this method is dependent on the level of parasitaemia and the technicians’ experience. Unfortunately, no other diagnostic methods are available. Once the presence of trypanosomes in blood and/or lymph is confirmed, a lumbar puncture has to follow for stage determination. The two parasites are morphologically identical and differentiation is only possible by analysis of isoenzymes and /or DNA-analysis.

The clinical presentation of Rhodesian and Gambiense sleeping sickness is remarkably different. The highly virulent *T.b. rhodesiense* causes a precipitated evolution of the disease (down to 8 days between infective bite and the presence of trypanosomes in the cerebrospinal fluid[5], while the less virulent *T.b. gambiense* about low parasitaemias, elusive and mild symptoms for months, and insidious evolution towards the nervous stage[6].

T.b. rhodesiense infections presents as an acute febrile illness that is fatal within weeks or months if left untreated[7]. At the site of the infective bite with *T.b. rhodesiense*, parasites proliferate and, occasionally, lead to nodule or ulcer called a chancre, involving widespread lymph node enlargement. It is very difficult to distinguish such symptoms in the beginning from other tropical fevers, such as malaria, bacterial meningitis or enteric fever. The parasite becomes established in the blood and it multiplies, with intermittent fever, splenomegaly and often signs of myocardial involvement. The study of the duration of symptoms and the case fatality of *T.b. rhodesiense* showed that the disease progressed to the stage of central nervous system involvement between three weeks to two months of infection. Compared to the Gambiense form, less demarcation between first and second stage illness is observed. The CNS involvement in *T.b. rhodesiense* infections can be clinically limited to drowsiness and tremor[8]. Most (> 80%) deaths occurred within six months of illness[9], often due to cardiac failure or secondary infections.

Clinical symptoms of Gambiense trypanosomiasis commence by an uncharacteristic general malaise. Typical but not characteristic signs and symptoms are fever, headache, joint pains, transient oedema, pruritus and lymphadenopathy may accompany the so-called first or haemolymphatic stage of HAT. Typical for *T.b. gambiense* HAT is the so called Winterbottom sign; enlarged, painless, rubbery cervical lymph nodes in the posterior cervical triangle. With the parasite's penetration to the central nervous system patients enter the second or meningoencephalitic stage which ends fatal when left untreated. The interval between the start of the infection and the start of the second stage is in the order of months or years[8] and allows *T.b. gambiense* patients to live up to 5 to 10 years post-infection. In the second stage of the disease neuropsychiatric signs and symptoms occur. Severe endocrinological and mental disturbances, such as impotence, infertility, amenorrhea, delirium, mania, paranoia, schizoid attacks, aggressive behaviour and severe motor problems are the main signs.

Until today, the underlying mechanisms of CNS invasion are not known in detail. Frequently observed is meningoencephalitis. The meninges are infiltrated with lymphocytes, plasma cells and occasional morular (Mott) cells. The inflammatory cell infiltrate extends along the Virchow-Robin spaces into the substance of the brain producing the characteristic picture of perivascular cuffing[10]. An immune response is certainly involved in the process; increased levels of antibodies can be detected and thus supports the inflammatory process. Further, the number of white blood cells (WBC) as well as the protein content is elevated in the cerebrospinal fluid (CSF). This observation is currently used for discrimination of first and late stage patients: arbitrarily, if more than 5 WBC per mm^3 and/or protein content above 25 mg per 100 ml (method of Siccard & Cantaloube) and/or trypanosomes are found in the CSF, the patient is considered to be late stage and treated with melarsoprol[3]. Recent investigations have indicated that the WBC count is more reliable than the protein determination[2].

3.2. Treatment of HAT

3.2.1. General Aspects

Only a very limited number of drugs are available for treatment of HAT and there is no vaccination. For first stage treatment two drugs are in use: suramin (Germanin™), a polysulphonated naphtylurea, applied in *T.b. rhodesiense* infection and pentamidine (Pentacarinat®), an aromatic diamidine, applied in *T.b. gambiense* infection.

Suramin was introduced into clinical use in 1920. It is normal clinical practice to start with a test dose to assess the patient's tolerance to the drug. Therefore a dose of 5mg/kg is given on the first day. For treatment of the early stage patients receive 5 doses of suramin at a dosage of 20mg/kg of body weight (up to a maximum of 1g). Because suramin has a terminal half-life of 92 days[11] each of the 5 doses is spaced by a 5 to 7 day interval. The major adverse drug reaction is nephrotoxicity with renal impairment. But also fever, joint pain, pruritus, exfoliative dermatitis, haemolytic anaemia, agranulocytosis, jaundice, hepatitis and diarrhea add to the adverse reactions profile. Suramin is a highly charged compound and does not cross the BBB. It is administered intravenously as a solution of suramin sodium. Due to instability to light the solution has to be freshly prepared before each administration. After intravenous administration it circulates in the blood in tight association with serum albumin and low density lipoproteins and is taken up slowly by host and parasite, probably by receptor mediated endocytosis. The different drug-uptake dynamics of host and parasite are believed to form the basis of the dissimilar toxicity profiles. Suramin is deposited in the renal tubes and should not be administered to patients with renal disease. Urine should be checked before and during treatment for proteinuria.

For treatment of the late stage of the disease there are no other registered drugs than melarsoprol and eflornithine. Melarsoprol, an organo-arsenical drug, was introduced by Friedheim in 1949. Eflornithine (α-Difluoromethyl ornithine, DFMO) was first developed as a cytostatic drug and was registered for the use against HAT in 1990 (USA) and 1991 (Europe)[12]. Eflornithine, which is active in *T.b. gambiense* infection has only very limited activity in *T.b. rhodesiense* due to biochemical reasons[13]. Melarsoprol, respectively its active metabolite melarsen oxide, is effective against both, *T.b. rhodesiense* and *T.b. gambiense* infections. For treatment of cases refractory to melarsoprol, nifurtimox is used on a compassionate base in combination with eflornithine. Nifurtimox, a 5-nitrofuran, is not registered against HAT, but was developed for the treatment of *Trypanosoma cruzi*, the causative agent of Chagas' disease.

Despite the search for new drugs and the introduction of eflornithine, melarsoprol is likely to remain the first-line drug for the next decade. For this reason WHO advocates optimization of it's use[2].

3.2.2. Melarsoprol Treatment

Treatment regimens of melarsoprol used to be empirically determined and vary considerably between countries and treatment centres. The treatment regimens for melarsoprol embody a real omnium-gatherum throughout Africa.

Melarsoprol treatment in *T.b. rhodesiense* patients is in general preceded by one or two injections of suramin. The pre-treatment is intended to clear parasitaemia in blood and lymph before the treatment

with melarsoprol starts. This is thought to prevent from an initial high antigen release which might trigger major adverse reactions in second stage treatment and the introduction of trypanosomes in the CNS while performing the diagnostic LP. However, there is no solid scientific evidence for this approach.

The current treatment schedules for melarsoprol in East Africa (see Table 1) are three or four series of daily i.v. injections (with increasing or equal doses up to a maximum dose of 3.6mg/kg) for 3 or 4 days, with intervening rest periods of 5-7 days. At the end of treatment the blood is checked for the absence of trypanosomes. Some health facilities also check the CSF at the end of treatment for the absence of trypanosomes and the WBC count. Cure can only be confirmed when the patients present for follow-up examinations, theoretically, after 3, 6, 12, 18 and 24 months. However, the drop-out rates in the follow-up period are very high and usually the patients come back for the first follow-up visit only.

Melarsoprol is a highly toxic drug and adverse drug reactions are frequent limiting the use of the drug to the late stage. The major adverse drug reaction in melarsoprol therapy is the potentially fatal encephalopathic syndrome (ES). The incidence for ES in Rhodesian sleeping sickness was found to be 8% and 5% in the Gambiense form[14], the mortality rate amongst those patients affected was approximately 50%. Until today, the mechanism of the ES is not fully understood. The current hypothesis proposes an immunological reaction[15]. In order to investigate this hypothesis more in detail, an association study was recently carried out: the occurrence of ES was correlated with the HLA-genotype of each patient. The HLA-system is highly polymorph and one of the major determinants of the immunological response. The alleles of the HLA-system are not only characteristic for each individual subject but also for different geographic regions. In a case-control design blood samples form *T.b. gambiense* patients were collected (Democratic Republic of Congo and Angola) and HLA-genotyping was performed. The association of the HLA-genotype and the incidence rates of ES showed that there is a possible correlation but the samples seize is yet too small[14].

Difficulties of current melarsoprol treatment in East Africa are various: the long hospitalization period overextends the health centres capacities; the changing dosages throughout the treatment for each individual patient are not accomplished regularly; wrong dosing can influence the frequency of adverse drug reactions and the case fatality rates.

The case fatality rates vary considerably between the different treatment centres. This reflects the local situation, e.g. doctors on site, availability of concomitant drugs, quality of the staff, nursing care for the patients etc.

The long hospitalization time presents a major burden for the patients and their families. The intermitted resting periods extends the hospital stay to up to one month or longer. This, together with the often long distances to the treatment centres lead to significant financial burden for the affected families. There are reports of patients leaving the health centres after one or two series of melarsoprol applications. Insufficient amount of treatment drugs favour relapses and resistances and hamper disease control on a long term basis.

Another problematic progression in melarsoprol treatment is an increasing number of refectory cases observed in Gambiense sleeping sickness. Growing number of patients, 20-25% in certain foci, do not respond to melarsoprol treatment, probably due to resistance[16]. Currently, two approaches are used to treat refractory cases: a) repetitive melarsoprol treatment, very often with limited success or b) the use combination treatment (eflornithine–nifurtimox & melarsoprol–nifurtimox). Clinical trials for testing

combination treatments are ongoing. Likely such a scenario will sooner or later also be required for treatment of *T.b. rhodesiense* in the lack of alternatives. Since eflornithine is not sufficiently effective in *T.b. rhodesiense* combinations with melarsoprol will be used. To achieve this, the abridged treatment schedule will be most helpful.

Recent reports by field workers about suramin resistance (unpublished) are a further concern. The parasites are exposed in first as well as in second stage treatment to suramin. Especially in second stage treatment very low doses are applied. The frequent uses of the drug at low doses may influence the development and dynamics of resistance.

3.2.3. Development of a new, abridged melarsoprol schedule against late stage infections

Even though melarsoprol was introduced in 1949 only recently its pharmacokinetic and pharmacological properties have been investigated[17-20]. Based on pharmacokinetics and computer simulations, a new schedule for late stage sleeping sickness was suggested. The alternative treatment schedule consists of a daily melarsoprol application at a dosage of 2.2mg/kg for 10 consecutive days. This leads to a reduction of the total amount of given drug of up to 30% and a reduced hospitalization time from about 30 to 14 days.

3.2.3.1. Safety, efficacy and effectiveness of the IMPAMEL schedule

Between 1996 and 2004 the safety, efficacy and effectiveness of the "Improved Melarsoprol Application – IMPAMEL" –schedule was clinically tested and developed.

The IMPAMEL I program assessed the safety and efficacy of this new, abridged treatment regimen by conducting an open, randomized equivalence trial in 500 *T.b. gambiense* patients in Angola. There were no significant differences in the frequency of adverse drug reactions and efficacy, and the new schedule was found to be favourable over the Angolan standard 26-day treatment schedule. The positive impression of the new treatment schedule was corroborated by the results of a multi-national, multi-centre study, monitoring the application of the new schedule in over 2'800 patients in various different settings (IMPAMEL II program) and the effectiveness was similar to the respective standard treatment regimens[21].

The IMPAMEL schedule doesn't improve the occurrence and/or frequency of adverse events which are related to the toxicity of melarsoprol. No significant clinical inferiority of the new schedule could be demonstrated. But the IMPAMEL schedule is very much favourable due to socio-economic benefits: with a similar efficacy and effectiveness over the standard regimens, the hospitalization time can be reduced by approximately 50%, the total amount of given melarsoprol by about 30%. Those factors facilitate late stage treatment on different levels: no more dose-adjusting, reduced hospitalisation time which relieves the health facilities, the patients and their families. Those factors have a high impact on the capacity of each treatment centre, on the treatment quality and compliance. Within the IMPAMEL II program a cost-effectiveness study was undertaken and the results confirmed that the new schedule reduces treatment and hospitalisation costs per patient.

On request of WHO, the new 10-day schedule for treatment of late stage *T.b. gambiense* sleeping sickness with melarsoprol was recommended by the International Scientific Council for

Trypanosomiasis Control and Research / ISCTRC at the occasion of the 27th meeting in October 2003, Pretoria, South Africa.

3.3. HAT Situation in East Africa

East Africa is affected by HAT in many different foci. Political unrest, civil wars and poverty result in large civil- and livestock movements which bear the potential of importing sleeping sickness to new areas. The establishment of control measures and interventions is very difficult and is hampered by the lack of financial means, reliable diagnostic tools, well trained personnel, access to health centres and by the high toxicity of the drugs in use. The HAT foci are spread over the rural areas where the population lives predominately from farming. Livestock is closely linked to their daily life; the close contact between humans and animals increases the risk of transmission. Game animals were the main reservoir in the past but today the domestic livestock (cattle and pigs) represent the most important animal reservoir for *T.b. rhodesiense*. The transmission from animals to humans takes place in the fields, cattle markets etc. Because of the high *T.b. rhodesiense* virulence, epidemics can quickly spread and cause substantial losses in human and economic sectors. Sleeping sickness has a considerable social and economic impact, with direct monetary losses plus indirect productivity losses (lost milk production, loss of market value). In Uganda, for example, the annual losses caused by *T.b. rhodesiense* are estimated at more than US$ 1.3 billion[22].

The existing diagnostic tools and treatment are very unsatisfactory. Most cases are diagnosed in the late stage of the disease only. Whether because the patients present at the health facility only after several visits to the traditional healers, or because the financial means were not sufficient for transport, or even because the detection of parasites was only confirmed after several visits in the health facilities. Another frequent reason for not seeking treatment in an early stage of the disease is the HIV epidemic: the local population believes that any sign or symptom of a disease is related to an HIV infection. There are multi-factorial reasons which can lead to a late diagnosis when melarsoprol remains the only possible treatment. The toxicity of melarsoprol causes many adverse drug reactions of which some can be fatal. The case fatality rates are in average around 9% but vary substantially between different treatment centres. The availability of concomitant treatments, the nutritional status of the patients, other infections and nursing care highly influences the case fatality rates.

The current treatment schedules in East Africa are very heterogeneous (Table 1). No standard treatment and care is established and a comparison is therefore not viable. Similarities of the treatment are found on the level of the suramin pre-treatment and the serial application of melarsoprol. Dissimilarities are found on the level of drug dosages, number of melarsoprol series, length of resting periods and concomitant treatments.

Another great difficulty of HAT epidemiology and control in East Africa is the high rate of under-detection. To formally quantify the level of under-detection of *T.b. rhodesiense* sleeping sickness during an epidemic (1988-1990) in Tororo (Uganda), a decision tree (under-detection) model was developed. It was calculated that for every reported death, 12 deaths remained unreported thus being equivalent to 92% of not reported deaths[23]. Uganda, Tanzania and Malawi report 50 to 1500 cases annually[24] but no quantification of cases can reflect the true impact of the disease. One can only say that the situation is very alarming.

3.3.1. Uganda

Uganda is the only country affected by both forms of the disease, *T.b. rhodesiense* in the South-East and *T.b. gambiense* in North-West of the country. Recently, the two closest foci in the centre of the country increased in size and approached each other significantly. A potential overlap of the two diseases has become possible and must be monitored carefully[25]. The two parasites are morphologically identical and only well equipped laboratories can distinguish the two parasites by animal infection, analysis of isoenzymes and/or DNA characteristics. Considering the capacities of the rural areas in Uganda, specific diagnosis of the two parasites is not feasible. *T.b. gambiense* can be sero-diagnosed with the CATT test (Card Agglutination Test), followed by microscopic confirmation. *T.b. rhodesiense* diagnosis can only be performed by microscopic techniques and is highly dependent on the level of parasitaemia and the technicians' experience. Treatments of the two forms of sleeping sickness differ in the first stage (pentamidine vs. suramin), as well as in the second stage (eflornithine vs. melarsoprol) of the disease. In case of a Gambiense-Rhodesiense overlap, combined with the lack of specific diagnosis, melarsoprol would immediately gain treatment priority because eflornithine is ineffective against *T.b. rhodesiense*. The indispensable need of treating each case with the same drug and according to the same schedule would quickly be recognized.

Uganda currently experiences a sleeping sickness outbreak which is ongoing since 1998. Large scale livestock movements for poverty reduction were undertaken. In the four years preceding the outbreak, the cattle population in the Soroti District grew by 660%[26]. The movement of *T.b. rhodesiense* infected cattle has resulted in new sleeping sickness foci in this District. The disease spread to the neighbouring Kaberamaido District, where more that 133,000 people have been put at risk of contracting the disease. The Ministry of Health supported the set up of a new treatment centre in Lwala, Kaberamaido District. Trainings and materials were provided by the Ministry of Health. From June 2004 until June 2005 approximately 205 patients were diagnosed and treated. Considering the last outbreak in Uganda that produced more than 4000 new cases in 1986[27] and the high rate of under-detection, the situation is startling and it is high time to implement relevant strategies in order to prevent (I) a further spread of the disease and (II) a possible overlap of the two disease zones.

3.3.2. Tanzania

Rhodesiense sleeping sickness, which was first reported in the 1920s and 1930s, was endemic in eight regions of Tanzania: Arusha, Kigoma, Lindi, Mbeya, Kagera, Rukwa, Ruvuma and Tabora. The disease tends to be concentrated in the North-West of the country, in Kigoma. In the past seven years only five regions have reported sleeping sickness cases; a large number originated in Kigoma (Kibondo and Kassulu districts).

Currently, between 4 and 5 million people are thought to be at risk of infection in Tanzania, but only 1% of these are under regular surveillance. In the past 30 years, the number of new cases reported annually has risen above 500 (Annual Sleeping Sickness Reports for Tanzania 1965-1995)[28].

The Tabora Station of the National Institute of Medical research (NIMR) supports all HAT activities in the area. Health centres in the region are sparse and most of them are not accessible throughout the year. For sleeping sickness, the Kaliua Health centre (Urambo District) has a good reputation and patients come from far to receive treatment. Kaliua has reported a very significant increase in number

of cases in 2004. Another problematic aspect in Tanzania were *T.b. rhodesiense* infections of several tourists, most of them contracted the disease in Game Parks[29]. The increased annual incidence of tourists in Tanzania further activated discussions about disease control and active surveillance.

A potential overlap of *T.b. gambiense* and *T.b. rhodesiense* sleeping sickness in North-West Tanzania is also possible, even if not as acute as in Uganda. As a consequence of the Great Lakes Crisis that has developed in Burundi and DRC, the International Federation of Red Cross and Red Crescent Societies (IFRC) have established refugee camps in the regions of Kasulu and Kigoma (1997). The Kigoma District received a massive influx of Congolese refugees. Especially refugees from DRC can potentially be infected with *T.b. gambiense*. Civil movement is ongoing and a potential overlap of the two diseases should be carefully observed.

3.3.3. Malawi

Out of the 25 districts there are 8 HAT-affected districts in Malawi: Rumphi in the northern region; Kasungu, Ntchisi, and Nhotakota in the central region; and Mangochi, Machinga, Chikwawa and Mulanje in the southern region[24]. Sleeping sickness in Malawi presents itself differently in comparison to the highly virulent and fulminate disease progression in other East African countries. Even though the parasite is clearly defined as *T.b. rhodesiense*, patients suffer from a chronic form of the disease as it is known from *T.b. gambiense* affected areas.

Diagnosis and treatment of sleeping sickness in Malawi is only provided at the District Hospitals. No regional health facility has the means and/or manpower to diagnose and treat sleeping sickness. Long geographical distances and difficulties in transport to the District Hospitals lead to very low numbers of passive case detection. Due to widespread poverty and lack of national priority there is no ongoing disease control.

In 2005, Malawi has joined the East African Network for Trypanosomiasis (EANETT). The primary aim of this collaboration is to commonly develop and support a National Control Program.

3.3.4. Situation in other East African countries

Kenya, Mozambique, Rwanda, Zambia and Zimbabwe report sporadic less than 50 cases annually. Botswana, Burundi, Ethiopia, Namibia and Swaziland have not reported any cases since 1990[24].

But large civil- and livestock movements can easily import *T.b. rhodesiense* to new areas, or historical foci can re-emerge.

3.3.5. Current Treatment Protocols in use in Eastern Africa

	UGANDA		TANZANIA		MALAWI		KENYA	
**Second stage treatment**	**Day of action**	**Dosage**	**Day of action**	**Dosage**	**Day of action**	**Dosage**	**Day of action**	**Dosage**
Suramin								
Application 01	1	5mg/kg	1	5mg/kg	1	5mg/kg		
Application 02			3	20mg/kg	2	20mg/kg		
Total Suramin		5mg/kg		25mg/kg		25mg/kg		
LUMBAR PUNCTURE (LP)	2		5		3		before treatment	
Melarsoprol								
Application 01	3	0.5mg/kg	5	2.2mg/kg	4	3.6mg/kg	1	3.6mg/kg
Application 02	4	0.72mg/kg	6	2.52mg/kg	5	3.6mg/kg	2	3.6mg/kg
Application 03	5	1.08mg/kg	7	2.88mg/kg	6	3.6mg/kg	3	3.6mg/kg
RESTING PERIOD	5 days		7 days		7 days		7 days	
Application 04	11	1.44mg/kg	15	2.88mg/kg	14	3.6mg/kg	11	3.6mg/kg
Application 05	12	1.80mg/kg	16	3.24mg/kg	15	3.6mg/kg	12	3.6mg/kg
Application 06	13	2.2mg/kg	17	3.6mg/kg	16	3.6mg/kg	13	3.6mg/kg
RESTING PERIOD	5 days		7 days		7 days		7 days	
Application 07	19	2.52mg/kg	25	3.6mg/kg	24	3.6mg/kg	21	3.6mg/kg
Application 08	20	2.88mg/kg	26	3.6mg/kg	25	3.6mg/kg	22	3.6mg/kg
Application 09	21	3.24mg/kg	27	3.6mg/kg	26	3.6mg/kg	23	3.6mg/kg
RESTING PERIOD	5 days		7 days				7 days	
Application 10	27	3.6mg/kg					31	3.6mg/kg
Application 11	28	3.6mg/kg					32	3.6mg/kg
Application 12	29	3.6mg/kg					33	3.6mg/kg
Total Melarsoprol		27mg/kg		28.08mg/kg		32.4mg/kg		43.2mg/kg
Total treatment days	13		11		13		12	
Total hospitalisation days	29		27		26		33	
Additional information								
Steroids	When reaction occurs		Prednisolone during treatment		Prednisolone or hydrocortisone during treatment		Hydrocortisone and saline during melarsoprol treatment	
LP on discharge	yes		No		yes		yes	
Follow up (in months)	passive 3,6,12,24		passive 3,6,12,18,24		passive 3,6,12,24		Active after 3months, passive 6,12,24	

3.4. Conclusion and Justification

The new, abridged protocol for melarsoprol[30] shows significant socio-economic benefits and better cost-effectiveness. Members of the Data and Safety Monitoring Board from the IMPAMEL I & II programs expressed their concerns regarding an interruption of the IMPAMEL program fearing it could lead to an uncontrolled use of the 10-day schedule in East Africa without reliable data about safety, tolerability and efficacy. Also, the WHO Scientific Working Group 2001 recommended the urgent conduct of the necessary clinical Phase II trials against *T.b. rhodesiense*, a call which was repeated by a WHO Afro meeting in Kampala, October 2003.

Like in *T.b. gambiense* treatments, there is a wide variety of East African treatment schedules in use: Kenya, Uganda, Tanzania and Malawi all follow different treatment protocols (Table 1). Similarities are the pre-treatment with suramin and the serial application of melarsoprol. Dissimilarities are found at the level of suramin and melarsoprol dosage, duration of resting periods and concomitant treatments. When implementing the short course in East Africa, several aspects have to be taken into consideration: Is the 10-day schedule safe and tolerable for *T.b. rhodesiense* patients? Is the pre-treatment with suramin redundant? Does the short course of melarsoprol have any impact on the incidence rate of the ES or the case fatality rates?

In the planning of any clinical program in *T.b. rhodesiense* sleeping sickness, the difficulties in patient's access and diagnosis and the overall limited total number of patients have to be kept in mind. All investigations have therefore to be limited to proof-of-concept studies. This clinical trial protocol covers two sequential proof-of-concept trials studies with total enrolment number of 60 patients.

One objective of the IMPAMEL III project is to investigate the importance of the suramin pre-treatment. Currently, the pre-treatment with suramin is administered for two reasons:

- To avoid the introduction of parasites into the CSF during the diagnostic LP.
- To clear parasites from blood and lymph in order to reduce the antigen release prior to melarsoprol administration.

The introduction of trypanosomes during LP is theoretically possible. However, the usage of proper materials and technical skills make a LP without blood vessel damage possible. In the rare case of trypanosome introduction to the CSF, the parasites are hampered in growth and survival as the CSF is a suboptimal medium[31]. Some authors believe that a high initial antigen release can trigger immunological overreactions[32], e.g. increase the risk of ES and consequently increase mortality. However, the dynamics of parasite elimination in the first hours after drug contact are very similar for melarsoprol and suramin[33]; the effect of the low-dosed suramin pre-treatment on the parasites is doubtful and will be assessed in these trials. Suramin pre-treatment policies are heterogeneous: in Uganda, only one test dose of 5mg/kg is administered; in Tanzania the test dose (5mg/kg) is followed by one full dose (20mg/kg). In Malawi the suramin pre-treatment is centre specific: some hospitals follow the same schedule as in Tanzania; others do not administer a suramin pre-treatment at all. Also Kenya's doesn't apply the pre-treatment with suramin anymore.

The parasite's exposure to the same drug in first as well as in second stage therapy is suboptimal. The insufficient doses used in the pre-treatment of late stage disease are rather prone to trigger drug resistances. Field reports of suramin treatment failures/resistances exist, but unfortunately no scientific data is currently available.

The additional use of suramin may also increase the risk of the patient for adverse drug reactions like renal impairment. During the IMPAMEL III trials, proteinuria will be monitored by urine analysis.

Suramin (Germanin™) is delivered in vials of 1g suramin sodium powder, which upon usage has to be dissolved in water and should be used within 15 minutes. Quantities remaining in the vial are not suitable for further use. The suramin pre-treatment tends to be uneconomic and the risk-benefit ratio should be re-considered.

The main objective of the IMPAMEL III trials is to assess the safety, tolerability and efficacy of the 10-day melarsoprol schedule against late stage *T.b. rhodesiense* sleeping sickness. The IMPAMEL schedule consists of 10 consecutive melarsoprol injections at a dosage of 2.2mg/kg. Currently, starting doses of national treatment schedules in Tanzania and Malawi are 2.2 mg/kg and 3.6 mg/kg, respectively. Only Uganda uses a lower starting dose of 0.36 mg/kg, increasing to a maximum dose of 3.6mg/kg with no better results regarding case fatality rates and/or ES reported. Late stage treatment according to the IMPAMEL schedule, reduces the total amount of given melarsoprol by 20% in Uganda, by 23% in Tanzania, by 33% in Malawi and by 50% in Kenya. Generally, there is evidence from former studies performed in *T.b. gambiense*[34] that the 10-day abridged treatment schedule leads to a comparable frequency of adverse drug reactions as the standard treatment schedule. The only exception may be skin reactions (like pruritus, maculopapular eruptions) or very rarely bullous reactions, which were observed in a higher frequency in previous trials.

The current national treatment policies are difficult and result in low treatment compliance, low treatment quality and very long hospitalization times: some of the patients leave the health facilities before the full melarsoprol course has been administered. This feature favours higher relapse rates and may have negative impacts on resistance development. The changing dosages throughout the treatment are rarely well implemented and can influence the adverse drug reactions profile. The long hospitalization times of the patients and their accompanying relatives reduces the overall capacity of the health facilities. The IMPAMEL schedule does not claim clinical superiority, but the socio-economic benefits and the better cost-effectiveness make it favourable to the patients, the health facilities and the national bodies of disease control. If treatment duration becomes shorter, treatment compliance and quality improve and may possibly also influence the health seeking behaviour. The IMPAMEL III project emphasizes awareness towards the disease, builds a good platform for capacity strengthening and supports the north-south collaborations.

Because the progression of *T.b. gambiense* and *T.b. rhodesiense* sleeping sickness is so different, each therapeutic intervention has to be tested separately. The patient's safety can only be ensured when possible differences in pharmacodynamics or –kinetics; or different susceptibilities to the same drug can be ruled out. Melarsoprol is a highly toxic compound, and until today, it is the only treatment for late stage *T.b. rhodesiense* patients. It is high time to test the 10-day melarsoprol schedule in East Africa: not only to be able to offer a substantial better treatment on the basis of an existing drug, but also to form the basis of a harmonization process of all East African treatment protocols in use. Most importantly, it can not be excluded, that melarsoprol will loose some of its efficacy against *T.b. rhodesiense*, similar to the phenomenon recently observed in some *T.b. gambiense* areas[35]. To test and potentially introduce the 10-day schedule in East Africa is a preparatory step for upcoming combination treatments as no alternative drug will be available in the next 5 to 8 years. For

pharmacological and practical reasons, all attempts for combination treatment with melarsoprol (with nifurtimox and eflornithine) are based on the abridged 10-day schedule.

The incidence rate of the encephalopathic syndromes (ES) under melarsoprol treatment is reported to be 8% in Rhodesiense and 5% in Gambiense sleeping sickness[14]. For about 50% of those cases the ES is a fatal event. According to current guidelines in drug safety, melarsoprol would never pass today's standards. Still today, little knowledge and data on the ES is available. In Angola and DRC, an association study was recently carried out where the ES was correlated with the HLA-genotype[14]. Unfortunately, the study could not provide enough samples in order to present significant results. Within the IMPAMEL III trials each patient is asked to provide a blood sample in order to investigate this correlation. Until today, no such data for *T.b. rhodesiense* is available.

HAT treatment requires a long-term follow-up of each patient in order to monitor drug safety and cure. Usually, patients are asked to present for follow-up visits after 3, 6, 12, 18 and 24 months. The drop-out rates are high, and on the occasion of the 9th September meeting, WHO formed a draft that reduces the follow-up period to one year (3, 6 and 12 months post-treatment). In most of the East African countries a maximum of one follow-up visit is recorded.

Some patients, who suffer from *T.b. rhodesiense* sleeping sickness, receive treatment and are then free of parasites. But only a few weeks later they can have trypanosomes again. Without well equipped laboratories it is not possible to classify such cases as relapses or re-infections. The IMPAMEL III trial includes a sub-study in order to distinguish relapses from re-infections: every patient is asked to provide a reference blood sample at baseline evaluation. In case of a positive follow-up result, an additional blood sample is collected. By comparison of the parasites genotype (PCR-Methodology), it is possible to distinguish relapses from re-infections.

A highly important aspect in HAT today is a potential overlap of *T.b. gambiense* and *T.b. rhodesiense* foci. The spread of *T.b. rhodesiense* is closely linked to civil-and livestock movements and often the political situations in the countries affected, lead to large numbers of displaced people. Large-scale livestock restocking activities are a common concept for poverty reduction. The sum of such actions can causes a potential spread of *T.b. rhodesiense*. Uganda has just experienced the establishment of a new *T.b. rhodesiense* focus, caused by large livestock movements. Uganda is affected by both forms of the disease. In order to monitor the potential geographical overlap, the IMPAMEL III project includes a separate blood sample analysis for each patient. Human infective *T.b. rhodesiense* is characterised by the presence of the so-called human-serum-resistance-associated-gene (SRA-gene)[36], which can be detected by conventional PCR.

The control of a zoonotic disease is very complex and costly: only an integrated approach of human and animal health can be an effective, long-term control strategy. With limited resources, priorities are set in specific and sensitive diagnosis and safe and effective treatment. However, the diagnostic tools of HAT are generally poor. The presence of trypanosomes can only be confirmed by microscopy but the two parasites are morphologically identical. A differentiated diagnosis, with the direct implementation of a differentiated treatment, is impossible. Standard treatment against first stage *T.b. gambiense* sleeping sickness is a 7-day pentamidine treatment, in first stage *T.b. rhodesiense*, suramin is used over a time period between 15 days (Malawi) to 31 days (Tanzania). For late stage therapy, melarsoprol is the standard treatment. However, cases refractory to melarsoprol exist already. Eflornithine, which has proven to be safer than melarsoprol[37] when it is properly administered,

is only active against *T.b. gambiense*. In case of an overlap of the two disease zones, which treatment will a late stage patient receive? How to treat mixed infections? To have at least the IMPAMEL schedule tested and implemented in both forms of the disease would greatly facilitate such a scenario.

As a conclusion, only the successful translation of knowledge and results gained on the level of trial sites to the national level will provide a significantly positive contribution to the patient's quality of life.

4. Objectives

4.1. Primary Objective

The primary objective is to further assess the safety, tolerability and efficacy of the 10-day melarsoprol schedule without suramin pre-treatment against late stage *T.b. rhodesiense* infections.

4.2. Secondary Objective

In a sub-study blood samples are collected in order to:

Continue the monitorig of the possible geographical overlap of *T.b. gambiense* and *T.b. rhodesiense* by analyzing all blood samples for the presence of the SRA-gene (PCR).

Distinction of relapses and re-infections in cases of positive follow-up results.

Investigation of a possible correlation between the HLA-genotype and the occurrence of the ES.

5. Investigational Plan

The trial protocol was developed in accordance with the Declaration of Helsinki and the ICH Guideline on Good Clinical Practice.

5.1. Overall Study Design

This is a two-centred, multinational utilization study. A minimum of 70 subjects with confirmed late stage *T.b. rhodesiense* sleeping sickness will receive 10 consecutive melarsoprol injections at a dosage of 2.2mg/kg (but a maximum of 180mg).

A minimum of 70 subjects who satisfy the inclusion and exclusion criteria will be enrolled in order to evaluate the primary efficacy endpoint (end of treatment). Enrolment is planned to begin in the second quarter 2007 and to be completed by January 2008. The last follow-up evaluation is expected to be completed approximately 17 months after the initiation of enrolment. Each patient is expected to participate for 12 months including the follow-up period.

The two trial sites are in Uganda and Tanzania:

Lwala Hospital, Kaberamaido District, Uganda

Lwala is close to Lake Kyoga and accessible by road throughout the year in a maximum 2 hours drive from Soroti. The hospital has 100 beds, the laboratory is sufficiently equipped and electricity is available from 7 to 10pm. The hospital us financed through a Diocese and also receives some government funds. . Only in the beginning of 2004 it has been established as HAT treatment centre, as a reaction to a new *T.b. rhodesiense* focus.

Kaliua Health Centre, Urambo District, Tanzania

Kaliua is accessible by road (in dry season only) in a maximum 3 hours drive from Tabora. The Kaliua Health Centre is a missionary hospital and has 50 beds. In the area of Tabora and Kigoma it has a very good reputation as HAT treatment centre. If needed, electricity is available. The infrastructure of the laboratory has been improved before the study initiation of the IMPAMEL III proof-of-concept trial.

Patient recruitment

Most of the patients are passively detected. In case of very low patient numbers active case search is planned high prevalence villages (for a time period of 3 to 6 days).

Also, referrals from other hospitals and health centres in the area are possible.

All subjects will undergo baseline microscopic blood examination to demonstrate the presence of trypanosomes. In case of a positive result, the diagnostic LP for stage determination (trypanosomes in CSF and/or WBC > 5 cells/mm^3) follows. Subjects will undergo safety evaluations including physical examination, adverse event monitoring, and collection of concomitant medications throughout the treatment period and at the end of treatment evaluation.

All subjects will undergo blood sampling and LP at baseline evaluation; end of treatment and at follow-up visits.

Children ≥ 6 years can be enrolled in all sites.

5.1.1. Efficacy

Primary efficacy will be evaluated in blood and CSF at the end of treatment (absence of trypanosomes). Secondary efficacy will be evaluated in blood and CSF (absence of trypanosomes) at each follow-up visit, 3, 6 and 12 months post-treatment.

Lumbar puncture will be performed at end of treatment and at all follow-up visits. In the case that trypanosomes and/or WBC > 5 cells/mm^3 are demonstrated, the patients will be re-treated according the centre policy.

In case of a positive blood test after the end of treatment or at follow-up visits, an additional blood sample will be taken (300µl, applied on Gentra Cards) to perform genotyping of the parasite (reference sample taken at baseline evaluation) and to define the case as a relapse or re-infection (PCR). Re-infections will not be considered as relapses or treatment failure.

Additional assessments of clinical efficacy will be performed at 12 month post treatment (last follow-up visit). The 12 month post-treatment evaluation is defined as the test of cure evaluation, based on the Draft Informal Consultation from WHO (9Sept2004).

5.1.2. Safety

The incidence of the encephalopathic syndrome (ES) with a fatal outcome and fatalities with other causes of death (e.g. disease related, opportunistic infections) have been set as combined endpoint of ≥10% (see 5.1.4).

5.1.3. Sub-Study

5.1.3.1. Blood sample collection for PCR-analysis

Patients are asked to provide 3, in case of a positive follow-up result, 4 blood samples. Blood sample analysis will provide knowledge on:

The possible strain-overlap in Tanzania and Uganda. The presence of the so-called SRA gene should be given in each sample.

At baseline evaluation, a reference blood sample from each patient will be taken. In case of trypanosome positive results during any follow-up visit, the genotyping of the parasites allows to make the distinction between relapses and re-infections.

Genotyping of the HLA-system will be put in correlation with the occurrence of ES[14]. At baseline evaluation a blood sample of each patient will be collected. Analysis will follow within 6 to 9 months after sample collection.

At any time during the study, the patient, or investigator, may elect to discontinue a patient's participation in the study.

5.1.4. Discussion of Study Design

The clinical data collected during the proof-of-concept trial gives evidence that (i) the suramin pre-treatment has no apparent positive impact on the treatment of late stage *T. b. rhodesiense* sleeping sickness and (ii) there are no atypical issues concerning the safety, tolerability and efficacy of the 10-day schedule. However, to provide statistical significant data it is necessary to increase the number of patients treated according this regimen. Considering the real-life situation in *T. b. rhodesiense* affected areas, it is most appropriate to further enrol and treat patients in a utilization study. Both trial sites have a long-standing experience with the treatment of late stage *T. b. rhodesiense* sleeping sickness and historic patient files are accessible. These files can be used as surrogate comparator to the IMPAMEL III data.

The utilization study will be conducted in the same trial sites as the proof-of-concept trial. Since study initiation of the proof-of-concept trial, both sites have gained a lot of experience and have become very competent collaborators. Furthermore, the multi-centre, multi-national approach allows to take the high strain heterogeneity of *T.b. rhodesiense* into account: data collection in two different endemic regions increases (I) the data's evidence and (II) reflects the general situation in East Africa in a more realistic

angle. Also, the respective countries are highly interested in the participation in the IMPAMEL III program. This is not only based on the acknowledgement of the objective's importance but also because the 10-day melarsoprol schedule forms the basis for any further development of treatment policies against second stage *T.b. rhodesiense* trypanosomiasis.

Diagnosis of trypanosomiasis and parasitological response to therapy will be assessed by the use of following tests: microscopic examination of blood (thin and/or thick smear), haematocrit centrifugation of blood (WOO)[38], microscopic examination of CSF fluid (single modified centrifugation) and WBC count.

The inclusion of children ≥ 6 years of age is considered as ethical and safe. Melarsoprol doses are calculated depended on body weight: a safe dose relation ship is best established using the individual body weight. Generally, the inclusion of children is a hot spot in international discussions on clinical trials. Today, the lack of information on pharmacokinetics and -dynamics in children is considered as unethical. Children are amongst the most vulnerable individuals and it is important to include children in the clinical development of new drugs and new therapeutic approaches. One sensitive topic to consider is that children can not give informed consent. Within the IMPAMEL III trials, parents will be asked to give consent for their children.

In order to reduce fever and pain, 1g of paracetamol will be administered twice a day. Paracetamol administration will start on the day patients have to undergo LP.

All trial patients will receive 10mg of prednisone half an hour prior to melarsoprol injection. Any other concomitant treatment, e.g. anticonvulsive drugs will be administered on a centre-specific basis and recorded in the Case Report Form.

Study efficacy parameters and timing of post-treatment evaluations are based on WHO Draft Informal Consultation on the Conduct of Clinical Trials in Human African Trypanosomiasis (9Sept2004). Historically, a 24 months follow-up period has been aimed for in order to confirm clinical cure. This draft guideline is developed for both, the chronic *T.b. gambiense* as well as the acute *T.b. rhodesiense* sleeping sickness. This has been barely achieved in practise. Especially the very fast progressing *T.b. rhodesiense* sleeping sickness causes serious clinical signs and symptoms within a few days or weeks after infection. The logistic burden of follow-up visits for the patients and the health facilities is not in balance with the therapeutic conclusions one can draw at the 24 month follow-up visit. The drop-out rate increases significantly after 6-12 month; approximately 60-80% of subjects will be lost to follow of by 18 months post-treatment. However, based on the WHO Draft Informal Consultation on the Conduct of Clinical Trials in Human African Trypanosomiasis (9Sept2004), the end of treatment has been chosen as primary endpoint and the follow-up visits as secondary endpoint.

The overall observed case fatality rate is approximately 9% and 10%, respectively according to the centre information (Lwala & Kaliua centres). The incidence of encephalopathic syndromes (ES) has been found to be in the order of 8% for Rhodesiense sleeping sickness with large inter-centre variations, of which about 50% have a fatal outcome. However, the infrastructure in field hospitals limits in-depth analysis of the cause of death, and it is therefore difficult to distinguish encephalopathic syndromes with a fatal outcome (drug related) and fatalities with other cause of death (e.g. disease related, opportunistic infections – not drug related). Therefore the endpoint for the stopping rule is set to ≥10% of combined fatal outcome of treatment. This includes the incidence of encephalopathic syndromes with a fatal outcome and fatalities with other cause of death (e.g. disease related,

opportunistic infections). For a group of 30 patients, this endpoint is equivalent to ≥7 patients. Final data analysis will be performed after study closure (Refer to Section 7.4).

Two trial sites will enrol subjects. Both trial sites are equipped with all necessary laboratory materials. Anti-trypanocidal drugs are available through WHO. For the supply of all other drugs, the STI will purchase the required amount needed for the IMPAMEL III trials.

The PCR-analysis for *T.b. rhodesiense* identification (SRA-gene) will be done in Uganda or Switzerland.

The PCR analysis for distinction of relapses and re-infections will be done at Yale University, USA.

5.2. Study Population

Both, male and female subjects ≥ 6 years who meet all the inclusion criteria listed in Section 5.2.1 and exhibit none of the exclusion criteria listed in Section 5.2.2 of this protocol, will be eligible for enrolment.

5.2.1. Criteria for Inclusion

The patient has second stage *T.b. rhodesiense* infection; i.e. parasitologically confirmed infection in the blood or trypanosomes in CSF and/or WBC > 5 cells/mm^3 in CSF detected by microscopic examination.

Patient is male or female and ≥ 6 years of age.

5.2.2. Criteria for Exclusion

The patient has first stage *T.b. rhodesiense* infection; i.e. presence of trypanosomes in blood upon microscopic examination and no trypanosomes in CSF and/or ≤ 5cells/mm^3.

Moribund or unconscious patients are excluded from the trial. The eligibility is evaluated following the Glasgow coma scale (see Appendix 1). Patients with less than 8 points are excluded from the trial.

Pregnant women are excluded from the trial.

Active clinical relevant medical conditions that in the Investigators opinion may jeopardize subject safety or interfere with participation in the study, including but not limited: significant liver disease, chronic pulmonary disease, significant cardiovascular disease, diabetes and open tuberculosis.

Critically ill patients with any condition which necessitates immediate and concomitant treatment not listed above.

The subject has been previously enrolled in the study.

5.2.3. HIV

On request, patients have the possibility to test their HIV status (in both trial sites HIV testing and counselling is normal practise).

HIV positive patients will be referred to the countries respective National Control Program in order to receive treatment.

HIV positive cases will be included in the trial when they meet all inclusion and exclusion criteria.

5.2.4. Criteria for Discontinuation

5.2.4.1. Discontinuation of Individual Subjects

A subject can be discontinued from the study for the following reasons:

1. Withdraws voluntarily from the study.
2. At the discretion of the Principal Investigator if the patient is not compliant to the requirements of the protocol.

If for any reason a subject is discontinued form the study before the end of treatment evaluation, the Investigator is required, as feasible, to perform the safety procedures planned for the end of treatment.

If a subject develops an ES, he/she will remain under observation until the adverse event is resolved and stabilized.

Subjects who discontinue the trial for any other reasons than drug or disease related reasons will be replaced (e.g. abandon of hospital, withdrawal of informed consent).

5.2.4.2. Discontinuation of the Entire Study

The entire study will be terminated if the combined endpoint (fatal ES and fatalities with other cause of death) is ≥10%, which corresponds to ≥ 7 patients in a group of 30 patients.

The STI may terminate this study prematurely; either for safety reasons, political unrests or census of false data. In the case of discontinuation of the entire study, the STI immediately notify the Investigator and subsequently provide written instructions for study termination.

5.2.4.3. Scientific Advisory Board

The study will be supervised by a SAB which supports and guides the IMPAMEL III program.

Members of the Scientific Advisory Board:

- Prof. Martin Schumacher, University of Freiburg, Germany
- Dr. Martin Odiit, MD, PhD, Uganda AIDS Commission, Kampala, Uganda
- Dr. Anne Moore, MD, PhD, Centre for Disease Control (CDC), Atlanta
- PD Dr. Blaise Genton MD, PhD, Swiss Tropical Institute, Basel, Switzerland

5.3. Treatment

5.3.1. Treatment schedule

The IMPAMEL III program aims at the further assessment of the safety, tolerability and efficacy of the 10-day melarsoprol schedule without suramin pre-treatment in late stage *T.b. rhodesiense* patients. In total, both trial sites will enrol a minimum of 70 patients. After ethical clearance is received, the study initiation will follow immediately. Best would be to start in both countries within close time periods in order to assure the availability of the results at similar time points.

Table 1: Trial schema

Day of treatment	Drug	Dose (mg/kg)
0	LP	--
1	Melarsoprol	2.2
2	Melarsoprol	2.2
3	Melarsoprol	2.2
4	Melarsoprol	2.2
5	Melarsoprol	2.2
6	Melarsoprol	2.2
7	Melarsoprol	2.2
8	Melarsoprol	2.2
9	Melarsoprol	2.2
10	Melarsoprol	2.2

The defined study population will be treated with melarsoprol at a dosage of 2.2 mg/kg (but a maximum of 180mg) for 10 days continuously. No suramin will be administered.

Table 2: Concomitant treatments during IMPAMEL III trials:

Concomitant treatments	
**Prednisone:**	Prednisolone (10mg) half an hour before melarsoprol administration.
**Paracetamol**	Patients (≥ 30kg): 1000mg every 12 hours<br>Patients (≤ 30kg): 250mg every 12 hours
**Anticonvulsive drugs**	centre-specific (best supportive care)
**Other drugs**	centre-specific (best supportive care)

Facilities participating in the study will exclusively use the schedule described for all patients who gave consent. In facilities not participating, the standard treatment schedule and supplementary medication as foreseen by the National Authorities will be used without modifications.

5.3.2. Additional Medication

If National rules exist they are applicable for additional medication and therapy. Any additional medication applied that deviates from the standard procedures in a respective centre must be listed in detail in the CRF (i.e. date of prescription, drug, duration, justification).

5.3.3. Drugs to avoid during the study

The use of thiabendazole should be strictly omitted because it is under suspicion of precipitating reactive encephalopathies[39].

5.3.4. 4.3.4. Supplementary medication before melarsoprol treatment (standard if no rules exist)

Malaria: centre specific diagnosis and treatment.

Helminthic infections: centre specific diagnosis and treatment.

Mebendazole: Day one to three: 100 mg total dose twice daily.

Paracetamol: before and after LP.

5.3.5. Supplementary medication during melarsoprol treatment

Each patient will receive paracetamol twice a day (every 12 hours). Patients (≥ 30kg) receive twice 1000mg; patients (≤ 30kg) will receive twice 250mg.

5.4. Procedure in case of severe adverse events

5.4.1. Encephalopathy

If preliminary signs of reactive encephalopathy are observed in a hospitalized patient (onset of fever and severe headaches) he has to be surveyed strictly. In case symptoms of reactive encephalopathy occur (convulsions, and/or confusion, coma) the melarsoprol treatment is interrupted. If no medical doctor is on site all efforts have to be made to get hold of him. All efforts have to be made that he is examined by a medical doctor immediately.

If reactive encephalopathy is diagnosed melarsoprol treatment is suspended and the patient is treated following the rules of IMC or any participating partner. The interventions below may serve as guidelines.

⇨ Immediate treatment in case of reactive encephalopathy:

- Adrenaline (epinephrine) may be given at 0.5 mg s.c. (to 1.0 mg max.) (0.5 ml of a 1:1000 solution, 1 mg/ml), the maximal dose is 4 mg per 24 hours.
- Corticosteroids (hydrocortison) at 1 g i.v., if the condition does not improve it may be repeated after some hours.

⇨ Additional treatment:

- No specific rules exist for the application of anticonvulsive drugs against encephalopathies exists. The given indication (diazepam 0.4 mg/kg i.v., then 0.2 mg/kg per hour added to an infusion fluid, if

possible) is based on [40], and glucose 5% or 10% infusions may be applied until the condition of the patient improves.

- The patient is to be kept under permanent surveillance and good general nursing until the condition improves, then the melarsoprol treatment can be resumed after 1 to 3 days. Treatment is resumed at the position where the encephalopathy has occurred. If two or less doses are missing the treatment will be considered complete and will not be resumed.

5.4.2. Other adverse events

5.4.2.1. Neuropathies

Neuropathies (sensitivity problems, severe pain when walking) are a phenomenon which occurs at largely different frequencies in different locations. If paraesthesias or severe pain occur which make an intervention necessary, the melarsoprol treatment is interrupted until improvement of the symptoms. Then treatment is resumed.

5.4.2.2. Skin Eruptions

In case skin eruptions (especially severe maculopapular eruptions or the begin of bullous lesions) (“exfoliative dermatitis”) occur the medical doctor has to be informed. A differential diagnosis is made in order to discriminate possible treatment related problems and concomitant infections.

The melarsoprol treatment is suspended if:

1. Lesions in the mouth or on any other mucous tissue are visible
2. Bullous lesions (exfoliative) are visible

An extended, confluent maculopapular rash occurs

After recovery the melarsoprol treatment is resumed with a third of the last used dose, at the following injection two thirds are applied, and then the full dose can be resumed. The patient has to remain hospitalized and kept under strict control. If two or less doses are missing the treatment will be considered complete and will not be resumed.

5.4.2.3. Hypotension or collapse

Collapse with slight hypotension:

0.3 - 0.5 mg Adrenaline (epinephrine) (0.3 - 0.5 ml, 1:000, 1 mg/ml) s.c.

Collapse with severe hypotension:

0.3 - 0.5 mg adrenaline (3 -5 ml, 1:10’000) very slowly i.v.; may be repeated after 5 - 10 minutes.

Complementary measures:

Macrodex 5%, Hydrocortison i.v. 100 -2000 mg every 6 hours, antihistamines (Diphenhydramine - type) 25 - 50 mg / 6h

5.5. Compliance

The local investigator of the executing organization or Authority is responsible for complete compliance with the investigators manual of all the medical doctors, nurses and staff involved.

- The investigator must agree and sign a document confirming that he or she understands the investigation and will work according to the protocol and Good Clinical Practice.
- The investigator is responsible for ensuring that the protocol is strictly followed. The investigator should not make any changes without the agreement of the STI, except when necessary to eliminate an apparent immediate hazard or danger to a subject.
- The investigator may take any steps judged necessary to protect the safety of the patients, whether specified in the protocol or not. Any such steps must be documented.
- During the treatment the records are maintained by the responsible nurse or the medical doctor. All entries have to be made clearly readable with a pen. The records are controlled by the medical doctor before discharge of the patient, where ever possible.
- The investigator must be thoroughly familiar with the properties, effects and safety of the investigational pharmaceutical product.
- Drugs are only applied by a medical doctor or a trained nurse assigned and adequately instructed. Exceptions in case of human resource constraints are allowed after discussion with the program director.

6. Study Plan

6.1.1. Pre-treatment Period

The subjects will be recruited (I) among the patients reporting to one of the trial sites and (II) during active surveillance in high prevalence villages.

The patients, who qualify for the study, will be asked to provide written informed consent prior to undergoing any study specific procedures or treatment.

For baseline evaluation following tests and procedures are to be performed:

Demographic information with height, weight, body mass index (calculated).

Complete medical history. For all women the fertility status will be noted (childbirth within 9 months, no birth within last 2 years, no birth within last 5 years or menopause).

Signs and symptoms typical of HAT will be required and evaluated, if present according to the HAT symptom grading scale outlined in Appendix 2: chancre, lymphadenopathy, malaise, general body pain, joint pains, headache, temperature, pruritus, cough, swelling of legs, dyspnoea, heart rate, diarrhea, nutritional status, daytime sleep, night time sleep, tremor, speech impairment, abnormal movements, walking disability, general motor weakness, unusual behaviour, inactivity, aggressivity, appetite and fertility (females only).

Concomitant diseases and pre-treatment medications for the 7-day period prior to doing.

Vital signs including blood pressure, heart rate and temperature, taken in sitting position.

Physical examination and Glasgow Coma Scale (Appendix 1).

Pregnancy test.

Confirmatory tests for presence of trypanosomes in blood and/or lymph, and lumbar puncture for stage determination.

The following tests will be performed in the order indicated to confirm the diagnosis of the screening examination with a maximum of 3 days prior to treatment initiation. Tests must be performed in order as indicated below; if a test is found to be positive for trypanosomes, subsequent tests need not to be performed:

Microscopic examination of blood (thin and/or thick smear).

Haematocrit centrifugation (WOO).

A sample of CSF (5ml) will be obtained by LP and the following tests will be performed:

WBC count for stage determination (average of 3 countings).

Modified single centrifugation technique[41]: microscopic examination of CSF for trypanosomes.

The subjects enrolled at a site will undergo following exams within 48 hours prior to dosing:

Blood sampling: for the performance of the tests listed, max. 5ml whole blood will be taken of each patient.

Haematocrit centrifugation (WOO).

Haematology (haematocrit, haemoglobin).

Quantifying the level of parasitaemia (thick blood film).

T.b. rhodesiense identification by PCR methodology (presence of SRA-gene).

Baseline data for each patient (blood collected on Gentra Cards). In case of a positive follow-up result, an additional blood sample will be taken. By comparison of the parasites genotype (PCR-Methodology) the distinction of relapse vs. re-infection is possible.

HLA genotyping (blood collected on Gentra Cards).

Subjects can require an HIV test (in the case of positive result they will be referred to the National Control Program). HIV positive patients can be included in the trial population.

Eligibility will be determined by the inclusion / exclusion criteria. Subjects are admitted as in-house patients to the clinical site for the entire treatment period.

Diagnosis and treatment of concomitant diseases such as malaria will be done according to current guidelines at the site before initiation of treatment with suramin. Treatment of filariasis will be postponed until completion of the application of trypanocidal drugs. Malaria will be tested on thin

and/or thick smear of blood. Any diagnosis will be recorded as a part of the Medical History and any medications prescribed to treat newly diagnosed diseases will be recorded.

6.1.2. Treatment Period

6.1.2.1. Study Drug administration

Melarsoprol will be given to the patient under direct observation of an authorized staff member. Patients receive 10 melarsoprol injections at a dosage of 2.2mg/kg, the injections are spaced by 24 hours.

Prednisone (10mg) is given to all trial patients half an hour before melarsoprol injection. Paracetamol will be given to the patients twice a day (every 12 hours). Patients with ≥ 30kg body weight will receive 2x1000mg and patients with ≤ 30kg body weight 2x250mg. In the mornings, paracetamol administration follows just after melarsoprol administration. 12 hours later the patients receive the second dose pf paracetamol.

Other concomitant treatments will be administered according to the centres guidelines (= best supportive care).

6.1.2.2. Procedures and Observations

Any concurrent medication taken during the treatment period will be recorded.

Any adverse signs and symptoms reported by the patient or noted during contact with the patient arising during the treatment period will be recorded. Refer to Appendix 4 for information regarding adverse event reporting requirements.

Vital signs will be recorded daily in the monitoring after each treatment dose.

6.1.3. End of Treatment

On study day 11 (after treatment) following tests will be performed in the order indicated:

Microscopic examination of blood (thin and/or thick smear).

Haematocrit centrifugation (WOO)

Haematology (haematocrit, haemoglobin)

Vital signs

In case of treatment failure:

Lumbar Puncture

Blood will be collected for the distinction of a possible relapse or re-infection (PCR).

Signs or symptoms of HAT will be queried and graded as outlined in Appendix 2: chancre, lymphadenopathy, malaise, general body pain, joint pains, headache, fever, pruritus, cough, swelling of legs, dyspnoea, heart rate, diarrhea, nutritional status, daytime sleep, night time sleep, tremor,

speech impairment, abnormal movements, walking disability, general motor weakness, unusual behaviour, inactivity, aggressivity, appetite and fertility (females only).

Concurrent medication taken during the study post treatment period (last day of study drug through discharge from the treatment centre) will be noted.

Any adverse signs and symptoms reported by the patient or noted during contact with the patient arising between the initial dose of study drug and end of treatment evaluation will be recorded. For the purpose of this study, disease progression and relapse will be considered as treatment failure, not as an adverse event. Refer to Appendix 4 for information regarding adverse event reporting requirements.

Before final discharge from the treatment centre, a physical examination, including vital signs and Glasgow Coma Scale, will be performed. Any deterioration in physical examination compared to the baseline examination should be reported as an adverse event.

All subjects and parents or guardians of adolescents will be educated to watch for potential adverse events that may develop after the patients is discharged from the treatment centre, including signs and symptoms of ES. Should adverse events present, the subject should immediately return to the treatment centre for evaluation.

6.1.3.1. Subject Long-Term Follow-up

The treating organization is responsible for the correct follow-up of the subjects. At the day of discharge each patient will receive a sleeping sickness treatment card that clearly indicated the dates of his/her follow-up visits. If support in transport is required, the IMPAMEL III program will cover the cost. In addition to the regular forms of the National Sleeping Sickness Program, the supplementary CRF forms provided for follow-up by STI must be used for every patient.

All subjects will undergo the following exams at the 3 month post treatment visit:

Microscopic examination of blood (thin and/or thick smear).

Haematocrit centrifugation of blood (WOO).

Haematology (haematocrit, haemoglobin).

Subjects will additional undergo a lumbar puncture. The WBC in the CSF will be counted. Microscopic examination of the CSF for trypanosomes will be performed (single modified centrifugation).

In case of positive results an additional blood sample will be taken for PCR- analysis in order to determine if it is a relapse or re-infection.

All subjects will undergo the following exams at the 6 month post treatment visit:

Microscopic examination of blood (thin and/or thick smear).

Haematocrit centrifugation of blood (WOO).

Haematology (haematocrit, haemoglobin).

Subjects will additional undergo lumbar puncture. The WBC in the CSF will be counted. Microscopic examination of the CSF for trypanosomes will be performed (single modified centrifugation).

In case of positive results an additional blood sample will be taken for PCR- analysis in order to determine if it is a relapse or re-infection.

All subjects will undergo the following exams at the 12 month post treatment visit:

Microscopic examination of blood (thin and/or thick smear).

Haematocrit centrifugation of blood (WOO).

Haematology (haematocrit, haemoglobin).

Subjects will additional undergo lumbar puncture. The WBC in the CSF will be counted. Microscopic examination of the CSF for trypanosomes will be performed (single modified centrifugation).

In case of positive results an additional blood sample will be taken for PCR- analysis in order to determine if it is a relapse or re-infection.

Signs or symptoms of HAT will be queried and graded as outlined in Appendix 2: chancre, lymphadenopathy, malaise, general body pain, joint pains, headache, temperature, pruritus, cough, swelling of legs, dyspnoea, hear rate, diarrhea, nutritional status, daytime sleep, night time sleep, tremor, speech impairment, abnormal movements, walking disability, general motor weakness, unusual behaviour, inactivity, aggressivity, appetite, fertility (females only).

At month 12 an oral interview and physical examination will be performed.

For the purpose of determining appropriate clinical evaluation and follow-up, subjects will be classified according to the following descriptions as favourable evolution, uncertain evolution, relapse, re-infection or death.

In case of suspicion of relapse, whole blood will be collected for PCR testing. Any adverse signs and symptoms which are spontaneously reported between the end of treatment evaluation and 30 days post-treatment will be recorded. The Investigator will report all serious adverse events, regardless of the time of the event relative to the completion of treatment. Refer to Appendix 4 for information regarding adverse event reporting requirements. The patient will be asked about his/her current health status (well or unwell). If the patient is not available at any post treatment evaluation, data about the status of the patient (alive and well or unwell; death and cause of death) will be gathered from family, friends or local authorities.

6.1.4. Monitoring for Safety

Treatment emergent adverse signs or symptoms will be recorded in the adverse event section of the Case Report Form, along with date(s) of occurrence, duration, degree of severity, and probable relationship to study drug. The observation time for adverse events starts when the treatment is

initiated and continues until discharge from the facility at the end of treatment. Any adverse signs and symptoms which are spontaneously reported between the end of treatment evaluation and 30 days post treatment will be recorded. The Investigator will report all serious adverse events, regardless of the time of the event relative to the completion of treatment. For the purpose of this trial, disease progression and relapse will be considered as treatment failure, not as an adverse event. Toxicity will be graded on a scale of 0 (no toxicity) to 4 using criteria in Appendix 3.

Table 3: Toxicity Grading and Actions Taken in Response to Toxicity

Toxicity	Action Taken
Grade 1:<br>Mild toxicity, usually transient, requiring no special treatment and generally, not interfering with usual daily activities	Observe patient closely and monitor laboratory parameters as needed. Patient may continue with study medication.
Grade 2:<br>Moderate toxicity ameliorated by simple manoeuvres	Observe patient closely and monitor laboratory parameters as needed. Patient may need to discontinue study medication.<br>If grade 2 toxicity is tolerated by patient, action same as grade 1.
Grade 3:<br>Severe toxicity which requires therapeutic intervention and interrupts usual activities; hospitalization may be prolonged.	NOTIFY STUDY COORDINATOR IMMEDIATELY.<br>Withhold test material. Monitor patient until event or toxicity decreases to Grade 2 or less. Discuss with study director whether to reinstitute treatment.
Grade 4:<br>Extremely severe or life-threatening	NOTIFY STUDY COORDINATOR IMMEDIATELY.<br>Discontinue test material treatment; monitor patient closely until event decreases to Grade 2 or less.

6.1.5. Collection and Reporting of Adverse Events

Instructions for definitions, collecting and reporting of adverse events are included in Appendix 4.

6.2. Efficacy and Safety Variables

6.2.1. Appropriateness of Measurements

Refer to Section 5.1. for discussion of the measurements to be used in the trial.

6.2.2. Primary Efficacy Variables

The primary efficacy variable will be the combined rate of clinical and parasitological cure (Table 5) at the end of treatment in the Per Protocol dataset (Section 6.1.3.). The combined rate of clinical and parasitological cure is defined as the proportion of treated subjects who have no clinical signs and symptoms of trypanosomiasis and no evidence for trypanosomes in any body fluid examined at all post treatment evaluations and not treated with other trypanosomiasis agent for any reason (early or late failure). At the end of treatment evaluation and at all follow-up visits lumbar puncture is performed and the cerebrospinal fluid (CSF) should contain ≤5 WBC/mm^3.

6.2.2.1. Secondary Efficacy Variables

Parasitological cure, clinical cure, relapse, re-infection and death rates at the 3, 6, and 12 month evaluations will be determined. Parasitological cure, relapse, re-infection and death rates will also be assessed at the 12 month test of cure evaluation. Clinical cure will be considered equivalent to the parasitological cure at the 12 month evaluation. Clinical Response Definitions, based on WHO Draft Informal Consultation are outlined in Table 5 (below). For purposes of statistical analyses, each patient will be defined within one of the following categories at each post treatment assessment, based on the appropriate characteristics.

Table 4: Clinical Response Definitions

Category	WHO Term	Patient Characteristics
Parasitological Cure	Cure	Lumbar puncture performed: No evidence for parasitological relapse and ≤ 5 WBC/mm^3 in CSF
Clinical Cure	Probable Cure	No evidence for parasitological relapse in absence of lumbar puncture (no clinical signs; symptoms / signs attributable to other disease; investigator decides no re-treatement necessary) **or**<br>No parasitological evidence of relapse with ≤ 5 WBC/mm^3 in CSF<br>**Action:** No re-treatment
Relapse	Relapse	Trypanosomes have been detected in any body fluid<br>**Action:** Parasite DNA analysis by PCR Methodology in order to distinguish relapse from re-infection – parasite genotype equal to original determination<br>Re-treatment
Re-infection		Trypanosomes have been detected in any body fluid<br>**Action:** Parasite DNA analysis by PCR Methodology in order to distinguish relapse from re-infection – parasite genotype different

		to original determination<br>Re-treatment
Death	Death	Death of patient during treatment or follow up; death will be categorized based on likely or definite cause of death as:<br>HAT<br>Adverse event related to treatment of HAT<br>Causes unrelated to HAT and treatment<br>Unknown causes

6.2.3. Safety Variables

The safety variables which will be evaluated through the end of treatment evaluation in this Phase II trial include: adverse events, vital sign measurements, laboratory results (blood, urine), physical examinations, and the use of any concomitant medications. Adverse events which are spontaneously reported between the end of treatment evaluation and 30 days post treatment will also be collected. The Investigator will report all serious adverse events, regardless of the time of the event relative to the completion of treatment.

7. Statistical Methods

7.1. Justification of Sample Size

The sample size of 2x30 subjects is not based on formal statistical analysis, but is regarded based on clinical judgment as sufficient in this Phase II trial to assess the safety and efficacy objectives of the study.

7.2. Efficacy Analysis

The primary efficacy endpoint is the parasitological cure 24 hours after completion of treatment. A secondary endpoint is the parasitological cure 3, 6 and 12 months after completion of treatment.

7.3. Safety and Tolerability Analysis

The primary outcome measure for safety is (I) death in temporal relationship to treatment. This includes the incidence of encephalopathic syndromes with a fatal outcome and fatalities with other cause of death (e.g. disease related, opportunistic infections). A combined endpoint of ≥ 10% has been set (N=30, CI=9.93 -42.28).

7.4. Stopping rule

Assuming a combined endpoint ≥ 10% of fatal outcome of treatment and a binomial distribution, then the probability of 7 or more events in 30 patients is approximately 0.026.

Therefore the trial would be stopped if ≥7 patients in a group of 30 patients would have a fatal outcome of their treatment. Final data analysis will be performed after study closure.

7.5. Statistical and Analytical Plan

The analytical plan will be developed during the execution of the study and added as an amendment to the protocol.

8. Protocol Deviations

When a variation from the protocol is deemed necessary for an individual subject, the investigator or other physician in attendance must contact the study coordinator (see Appendix 4).

Such contact must be made as soon as possible to permit a decision as to whether or not the subject is to continue in the study. The deviation from the protocol will be authorized only for that subject.

All deviations related to study inclusion and exclusion criteria and significant deviations to subject management and protocol procedures must be documented on the appropriate case report form.

Changes to the protocol (after Signatures of Agreement are obtained) that affect the decision of the Ethics Committee (e.g., more extensive procedures, increased risk to subjects, changes in the subject population, additional safety information, etc.) must be documented in the form of an amendment. This amendment must be signed by the appropriate STI personnel and the Investigator, and approved by the Ethics Committee before it may be implemented. If the amendment is minor or reduces the risk to the subject, the chairperson of the Ethics Committee alone may approve it. Ethics Committee acceptance is not necessary for protocol clarifications that consist of minor protocol changes such as correcting typographical errors, rewording for clarity, changes in monitoring personnel, or for other changes to the protocol that do not affect the conduct of the study, including changes in the plan for statistical analysis.

The only circumstances in which the amendment may be initiated without Ethics Committee acceptance is where the change is necessary to eliminate apparent immediate hazards to the subjects. In that event, the Investigator must notify the Ethics Committee in writing within five (5) working days after the implementation.

9. Ethics and Regulatory Requirements

9.1. Ethical Clearance

The study protocol was developed in accordance with the Declaration of Helsinki and the ICH Guidelines on Good Clinical Practice.

Before study initiation the protocol must be approved by the Ethics Committee of the Kanton beider Basel (EKBB) and the Ethics Committee of the Ugandan Ministry of Health and the Tanzanian National Institute for Medical Research (NIMR).

9.2. Ethical Conduct of the Study

The IMPAMEL III trial is registered in the Current Controlled Trials (CCT, sister company of Biomed Central) database, which allow users to search, register and share information about clinical trials. Registration Number is ISRCTN40537886.

The study will be conducted in accordance with the protocol, all ICH and GCP regulations governing clinical study conduct; ethical principles that have their origin in the Declaration of Helsinki (Appendix 5), and all applicable local laws and regulations. The investigator must assure that the study is conducted complies with prevailing local laws and customs. Responsibilities of the Investigator are specified in Appendix 5.

9.3. Subject Information and Consent

Patients will receive the information on the benefits and risks of the treatment and will be asked for consent to get treatment. Every patient is given the choice to participate in the trial or to receive the standard treatment. For inclusion of children, the parents (or guardian or legal representative) are asked to sign the Informed Consent.

9.4. Subject Confidentiality

The investigators must ensure that the subject's anonymity will be maintained. Subjects will be identified on the Case Report Forms by the Subject Number and Subject's Initials in addition to centre and study identification information. The investigators will keep a separate confidential enrolment log that matches identifying codes with the subjects' names and residencies.

10. Other Administrative and Regulatory Procedures

10.1. Data Quality Assurance

The National Coordinates are responsible for an adequate data quality.

Prior to the initiation of the study, an investigator's meeting will be held with the investigators and their study coordinators and staff from STI. This meeting will include a detailed discussion of the protocol, performance of study procedures, CRF completion, and specimen collection methods. In addition to the investigator's meeting, the study personnel at each site will be trained on the study procedures by STI personnel at a study initiation visit.

STI personnel will monitor each site throughout the study. Source document review will be performed against entries on the CRF and a quality assurance check will be performed to ensure that the investigator is complying with the protocol and regulations. In addition, after CRFs are retrieved by the STI personnel. A review of the data will be conducted by a physician and a clinical review team at STI.

All data hand entered in the database will be verified by a double-key entry procedure. Any discrepancies will be reviewed against the hard copy CRF and corrected on-line. After completion of the entry process, computer logic checks will be run to check for such items as inconsistent study dates and outlying laboratory values. Any necessary corrections will be made to the database and documented via addenda or audit trail. A manual review of selected line listings will also be performed at the end of the study.

10.2. Source Documents, Case Report Forms (CRFs)

All CRFs will be controlled for completeness locally. The data will be double-entered into a DMSys database prepared by the study coordinator. The quality of the final database is a subject to auditing by the PMU.

Subject source documents are the physician's subject records maintained at the study site. In most cases, the source documents will be the hospital's or the physician's chart. In cases where the source documents are the hospital's or the physician's chart, the information collected on the CRFs must match those charts. In some cases, a portion of the source documents for a given subject may be the CRFs.

The CRFs will be printed on CR ("carbon required") paper to permit multiple copies. The bottom copy is to be retained at the site for the Investigator's study file. All questions should be answered using a black ink ballpoint pen. If certain data are not available, not done, or not applicable: "NAV," "ND," or "NAP," respectively, will be entered in the appropriate space.

Twenty-four hour clock should be used for all time entries. Changes and/or additions to data entered on original CRFs must be made in the following manner: The original entry will be lined out with a single line drawn through the error (not erased or "whited out") so as to leave it still legible. The correction will be entered using a black ink ballpoint pen, initialled, and dated by the person making the correction. The Investigator or delegate (e.g., Sub-Investigator or study coordinator), may enter corrections on original CRFs. The monitoring team may make changes to the copies of CRFs based on information supplied by the Investigator and documented in the study file.

An STI staff member or assigned consultant will bring the CRFs to the STI.

An STI staff member or assigned consultant will visit the study site for the purpose of comparing the data on the CRFs with the source documents. The Investigator agrees to make source documents available for this purpose.

The CRFs should be completed as soon as possible after the data are available.

10.3. Maintenance of Records

The Investigator will maintain all study documents:

a. for a minimum of two years after study closure.

OR

b. for any longer period that is specified by the regulatory requirements of the country in which the study site is located.

If the Investigator relocates, retires, or for any reason withdraws from the study, the study records may be transferred to an acceptable designee, such as another Investigator, another institution, or to STI. The Investigator must obtain STI's permission before disposing of any records.

It is the responsibility of the STI to inform the investigator/institution as to when these documents no longer need to be retained.

10.4. Completion of the Study

The investigator will conduct this study in compliance with the protocol, and will complete the study in satisfactory compliance with the protocol within 8 weeks after the last evaluation of the last subject or

within 8 weeks of the designated completion date. The investigator will provide a summary of the study's outcome to the IEC and the STI.

10.4.1. Final Report

Upon request by the Investigator, at the completion of the study and following analysis of the data, STI will supply a listing of the Investigator's subjects' treatment assignments, tabulated data, and statistical analyses, as appropriate. A copy of the final study report and corrected CRFs and/or change requisitions including a receipt to be signed and returned to STI will be provided to each Investigator following its release.

10.4.2. Use of Information

All information concerning the IMPAMEL III project belongs to the STI and is confidential (e.g. basic scientific data, or and not previously published is considered confidential information).

The information developed during the conduct of this clinical study is also considered confidential and will be used by the STI. To allow for the use of the information derived from this clinical study and to ensure complete and thorough analysis, the investigator is obligated to provide STI with complete test results and all data developed in this study and to provide direct access to source data/documents for trial-related monitoring.

10.5. Publication

The final results of the investigations will be published in scientific journals and at seminars or conferences.

All results from investigations are considered confidential and shall not be made available to any third part by any member of the investigating team before publication.

A restrictive definition of authorship of any publications will be applied:

Any author must have made significant contributions to (a) the conception and design, or analysis and interpretation of the data; *and* to (b) drafting the article or revising it critically for important intellectual content; *and* on (c) final approval of the version to be published. Each author must have participated sufficiently in the work to take public responsibility for the total content of the publication.

The guidelines follow recent publication regarding this issue[42].

The study coordinator will be the first author of the main scientific papers to be published. Country coordinators will be considered as co-authors for publications containing data from their countries, provided a continuous active participation throughout the conduct of the project, e.g. from drafting the study protocol to the writing of the manuscript is warranted.

10.6. Country Specific Investigator's Signature Page

1. I have read this protocol and agree to conduct the study as outlined and in accordance with all applicable local, state, and federal regulations.
2. I agree to maintain the confidentiality of all information received or developed in connection with this protocol.

____ ________________

Signature of Local Principal Investigator Date

____

Printed Name of Local Principal Investigator

Appendix 1 Glasgow Coma Scale (adapted)

Adults	Reaction	Score
Best verbal response	Oriented	5
	Confused	4
	Inappropriate words	3
	Incomprehensive Sounds	2
	None	1
Best Motor Response	Obeys Commands	6
	Directed Defensive Response	5
	Non-directed Defensive Response	4
	Flexion to Pain	3
	Extension to Pain	2
	None	1
	Total	2-11
	Considered unrousable coma	≤ 7

Appendix 2 Grading of Clinical Signs and Symptoms of HAT

	Grade 0	Grade 1	Grade 2
Chancre	absent	present	
Lymphadenopathy	absent	palpable (> 1 cm)	
Malaise	absent	present	unbearable
General body pain	absent	present	unbearable
Joint pains	absent	present	unbearable
Headache	absent	present	unbearable
Fever	absent	> 37.5ºC	
Pruritus	absent	present	visible traces of scratching
Cough	absent	non-productive	productive
Swelling of legs	no swelling	swelling limited to foot	Swelling whole leg
Dyspnoea	none or no change	on exertion	at rest
Heart rate	regular	non-regular	
Diarrhea	absent	3 stools in the last 24 hours	More than 3 stools in the last 24 hours
Hepatomegaly	absent	present	severe
Splenomegaly	absent	present	severe
Nutritional Status	normal	suboptimal	malnourished
Daytime sleep	normal	repeatedly	continuously
Night time sleep	normal	few hours	rare
Tremor	absent	visible	severe
Speech impairment	absent	present	uninterpretable speech
Abnormal movements	absent	present	inability to perform daily tasks
Walking disability	absent	walking with difficulties	walking with help<br>or inability to walk
General motor weakness	absent	ability to stand up from chair without use of hands	no ability to stand up from chair without use of hands
Unusual behaviour	absent	present	severe
Inactivity	absent	reduced workforce	inability to perform daily tasks
Aggressive behaviour	absent	sporadic	severe, requires observation
Appetite	normal	disturbed	severely disturbed
Fertility (females only)	Birth within last 9 months	No birth within last 2 years	No birth within last 5 years or menopause
Breast-feeding(females only)	absent	present	

Appendix 3 Toxicity Grading Scale

WHO (World Health Organization) Toxicity Criteria by Grade

Category	Toxicity	Grade 0	Grade 1	Grade 2	Grade 3	Grade 4
Haematology	WBC ($x10^3$/l)	> 4	3.0 - 3.9	2.0 - 2.9	1.0 - 1.9	< 1.0
Haematology	Hct(%)	> 40-54% (for men), > 37-47% (for women)	> 39% (for men), > 36% (for women)	<39% (for men), < 36% for women	< 32% (for men), < 29% for women	< 25% (for men), < 22% (for women)
Haematology	HGB (g/100ml)	WNL	10.0 normal	8.0-10.0	6.5-7.9	<6.5
Metabolic (fasting)	Hyperglycaemia (mg/dl)	< 116	116 - 160	161 - 250	251 - 500	> 500 or ketoacidosis
Metabolic (fasting)	Hypoglycaemia (mg/dl)	> 64	55 - 64	40 - 54	30 - 39	< 30
Gastrointestinal	Nausea	None	able to eat reasonable intake	intake significantly decreased but can eat	no significant intake	__
Gastrointestinal	Vomiting	None	1 episode in 24 hrs	2 - 5 episodes in 24 hrs	6 - 10 episodes in 24 hrs	> 10 episodes in 24 hrs or requiring parenteral support
Gastrointestinal	Diarrhoea	None	increase of 2 - 3 stools / day over pre-Rx	increase of 4 - 6 stools / day, or nocturnal stools, or moderate cramping	increase of 7 - 9 stools / day, or incontinence, or severe cramping	increase of > 10 stools / day or grossly bloody diarrhoea, or need for parenteral support
Gastrointestinal	Stomatitis	None	painless ulcers, erythema, or mild soreness	painful erythema, oedema, or ulcers but can eat solids	painful erythema, oedema, or ulcers and cannot eat solids	requires parenteral or enteral support for alimentation
Liver	Liver- clinical	No change from baseline	-----	-----	Pre-coma	hepatic coma
Kidney, bladder	Proteinuria	No change	1 (+) or < 0.3 g% or 3 g/L	2 - 3 (+) or 0.3 - 1.0 g% or 3 - 10 g/L	4 (+) or > 1.0 g% or > 10g/L	nephrotic syndrome
Kidney, bladder	Haematuria	Negative	microscopic only	gross, no clots no Rx needed	gross and clots bladder irrigation	requires trans-fusion or cystectomy
Pulmonary	Pulmonary	none or no change	asymptomatic, with abnormality in PFTs	dyspnoea on significant exertion	dyspnoea at normal level of activity	dyspnoea at rest

Category	Toxicity	Grade 0	Grade 1	Grade 2	Grade 3	Grade 4
Cardiac	Cardiac arrhythmias	none	asymptomatic, transient, requiring no therapy	recurrent or persistent, no therapy required	requires treatment	requires monitoring; or hypo- tension, or ventricular tachycardia or fibrillation
Neurologic	Neuro: sensory	none or no change	mild paraesthesias; loss of deep tendon reflexes	mild or moderate objective sensory loss moderate paraesthesias	severe objective sensory loss or paraesthesias that interfere with function	-----
Neurologic	Neuro: motor	none or no change	subjective weak- ness; no objective findings	mild objective weakness without significant impairment of function	objective weak- ness with impairment of function	paralysis
Neurologic	Neuro: cortical	none	mild somnolence or agitation	moderate somnolence or agitation	severe somnolence, (>50 % waking hours), agitation, confusion, disorientation or hallucinations	coma, seizures, toxic psychosis
Neurologic	Neuro: cerebellar	none	slight incoordination, dysdiadochokinesia	intention tremor, dysmetria, slurred speech, nystagmus	locomotor ataxia	cerebellar necrosis
Neurologic	Neuro: mood	no change	mild anxiety or depression	moderate anxiety or depression	severe anxiety or depression	suicidal ideation
Neurologic	Neuro: headache	none	mild	moderate or severe but transient	unrelenting and severe	-----
Neurologic	Neuro: constipation	none or no change	mild	moderate	severe	ileus > 96 hrs
Neurologic	Neuro: hearing	none or no change	asymptomatic, hearing loss on audiometry only	tinnitus	hearing loss interfering with function but correctable with hearing aid	deafness not correctable

Neurologic	Neuro: vision	none or no change	-----	-----	symptomatic subtotal loss of vision	blindness
Pain	Pain	none	mild	moderate	severe	reg. narcotics
Skin	Skin	none or no change	scattered macular-papular eruption or erythema that is asymptomatic	scattered macular or papular eruption or erythema with pruritus or other associated symptoms	generalised symptomatic macular, papular or vesicular eruption	exfoliative dermatitis or ulcerating dermatitis
Allergy	Allergy	none	transient rash, drug fever < 38°C (100.4°F)	urticaria, drug fever 38°C (100.4°F), mild bronchospasm	serum sickness, bronchospasm requiring parenteral medication	anaphylaxis
Local	Local	none	pain	pain and swelling with inflammation or phlebitis	ulceration	plastic surgery indicated
Fever of unknown origin	Fever of unknown origin	none	37.1 - 38.0°C 98.7 - 100.4°F	38.1 - 40.0°C 100.5 - 104°F	> 40.0°C (> 104.0°F) for less than 24hrs	> 40.0°C (>104°F) for more than 24 hrs or accompanied by hypotension
Infection	Infection	none	mild	moderate	severe	life-threatening

Appendix 4 Administrative Procedures for the Reporting of Adverse Events

Adverse Event

The Investigator will be required to provide appropriate information concerning any findings that suggest significant hazards, contraindications, side effects, or precautions pertinent to the safety of the treatment schedule under investigation.

Types of adverse events

The term adverse event could include any of the following events which develop or increase in the severity during the course of the study:

Any signs or symptoms whether thought to be related to the condition under study;

Any abnormality detected during physical examination.

These data will be recorded on the appropriate CRFs, regardless of whether they are thought to be associated with the study or the drug under investigation. Associated with the use of the drug means that there is a reasonable possibility that the event may have been caused by the drug, respectively by the treatment schedule.

Adverse signs or symptoms will be graded by the Investigator as mild, moderate, severe, or intolerable according to the following definitions:

Grade	Definition
(1) Mild:	Causing no limitations of usual activities
(2) Moderate:	Causing some limitations of usual activities
(3) Severe:	Causing inability to carry out usual activities
(4) Intolerable:	Intolerable or life-threatening

The observation time for adverse events starts when the treatment is initiated and continues until 5 days post-treatment. Any adverse signs and symptoms which are spontaneously reported between the end of treatment evaluation and 30 days post treatment will be recorded. For the purpose of the trial, disease progression and relapse will be considered as treatment failure, not as an Adverse Event. Cases of re-infection will not be considered as treatment failure.

Relationship to the Study Drug

The investigator will use the following definitions to assess the relationship of the adverse event to the use of the study drug:

**Probably related**	An adverse event has a strong temporal relationship to study drug, or recurs on re-challenge and etiology is unlikely or significantly less likely.
**Probably not related**	An adverse event has a strong temporal relationship to the study drug and an alternative etiology is equally or less likely compared to the potential relationship to the study drug.
**Possibly related**	An adverse event has little or no temporal relationship to the study drug and/or more likely alternative etiology exists.
**Not related**	An adverse event is due to an underlying or concurrent illness or effect of another drug and is not related to the study drug (e.g. has no temporal relationship to study drug or has much more likely alternative etiology.

If an investigator's opinion of possibly, probably not, or not related to study drug is given, an alternative etiology must be provided by the investigator of the adverse event.

Serious Adverse Event

A "serious" adverse event is defined as any event that suggests a significant hazard, contraindication, side effect, or precaution. A serious adverse event includes any event that:

is fatal

is life threatening, meaning, the subject was, in the view of the Investigator, at immediate risk of death from the reaction as it occurred, i.e., does not include a reaction that, had it occurred in a more serious form, might have cause death;

is a persistent or significant disability or incapacity, i.e., the event causes a substantial disruption of a person's ability to conduct normal life function;

requires, or prolongs in-patient hospitalization;

is a congenital anomaly or birth defect;

Is an important medical event, based upon appropriate medical judgment that may jeopardize the patient or subject or may require medical or surgical intervention to prevent one of the other outcomes defining serious.

Reporting Obligations

All “serious” events, whether or not unexpected or considered to be associated with the use of drug, and regardless of the timing of the occurrence after the first dose of study drug, must be communicated immediately upon discovery of the event, and within 24 hours of discovery, either by telephone or email.

Contact: Study coordinator
Irène Kuepfer
Swiss Tropical Institute
Socinstrasse 57
CH-4002 Basel,
Fax +41 61 225 26 78
Tel +41 61 225 26 68
Email: Irene.Kupefer@unibas.ch

Follow-up of adverse events

All Serious Adverse Events must be followed with appropriate medical management until resolved or until considered chronic and stable or otherwise explained. If the treatment was interrupted due to an adverse event, it may be resumed if considered both safe and ethical. The minimum coherent duration of treatment must be maintained if the patient is re-treated.

Appendix 5 Ethical Consideration and Human Subject Protection

Investigators and Study Sites

The investigators who are responsible for the conduct of this study, in compliance with this protocol, are identified on the Signatures of Agreement page.

Ethics Committee Acceptance

It is required that a valid Ethics Committee approves in writing the conduct of this clinical study, together with the Investigator's informed consent document, prior to study initiation.

The trial protocol was developed in accordance with the Declaration of Helsinki and the ICH Guidance on Good Clinical Practice.

In performing this study, both the Investigator and Sponsor endorse, as a minimum, the standards for conduct of clinical research activities as set forth in the Declaration of Helsinki, ICH guidelines and local country laws and regulations.

The Study Director will submit the protocol and informed consent for Ethics Committee acceptance. This will be appropriately documented. The Ethics Committee should be asked to give its acceptance in writing. The names and qualifications of the members of the review committee will be recorded and submitted to the Swiss Tropical Institute together with the written acceptance for the conduct of the study.

The members of the Ethics Committee accepting must be independent of the sponsor and the Investigator.

The written acceptance should consist of a completed Ethics Committee Acceptance form or written documentation from the Ethics Committee containing the same information.

Until written acceptance by the Ethics Committee has been received by the Sponsor, no subject may undergo any procedures solely for the purpose of determining eligibility for this study.

Protocol amendments must also be reviewed and accepted by the Ethics Committee and written acceptance from the committee or at least the chairperson (or a designated committee member) must be received by the Swiss Tropical Institute before implementation. This written approval will consist of a completed Ethics Committee Acceptance form or written documentation from the Ethics Committee containing the same information.

Additional, the IMPAMEL III trial is registered in the Current Controlled Trials (CCT, sister company of Biomed Central) database, which allows users to search, register and share information about clinical trials.

Declaration of Helsinki

World Medical Association Declaration of Helsinki: Recommendations Guiding Physicians in Biomedical Research Involving Human Subjects

Adopted by the 18th World Medical Assembly, Helsinki, Finland 1964 and as revised by the World Medical Assembly in Tokyo, Japan 1975, in Venice, Italy, 1983, and Hong Kong in 1989.

Introduction

It is the mission of the physician to safeguard the health of the people. His or her knowledge and conscience are dedicated to the fulfilment of this mission.

The Declaration of Geneva of the World Medical Association binds the physician with the words, "The health of my patient will be my first consideration," and the International Code of Medical Ethics declares that, "A physician shall act only in the patient's interest when providing medical care which might have the effect of weakening the physical and mental condition of the patient. "

The purpose of biomedical research involving human subjects must be to improve diagnostic, therapeutic and prophylactic procedures and the understanding of the etiology and pathogenesis of disease.

In current medical practice most diagnostic, therapeutic or prophylactic procedures involve hazards. This applies especially to biomedical research.

Medical progress is based on research which ultimately must rest in part on experimentation involving human subjects. In the field of biomedical research a fundamental distinction must be recognised between medical research in which the aim is essentially diagnostic or therapeutic for a patient, and medical research the essential object of which is purely scientific and without implying direct diagnostic or therapeutic value to the person subjected to the research.

Special caution must be exercised in the conduct of research which may affect the environment, and the welfare of animals used for research must be respected.

Because it is essential that the results of laboratory experiments be applied to human beings to further scientific knowledge and to help suffering humanity, the World Medical Association has prepared the following recommendations as a guide to every physician in biomedical research involving human subjects. They should be kept under review in the future. It must be stressed that the standards as drafted are only a guide to physicians all over the world. Physicians are not relieved from criminal, civil and ethical responsibilities under the law of their own countries.

I. Basic Principles

1. Biomedical research involving human subjects must conform to generally accept scientific principles and should be based on adequately performed laboratory and animal experimentation and on a thorough knowledge of the scientific literature.

2. The design and performance of each experimental procedure involving human subjects should be clearly formulated in an experimental protocol which should be transmitted to a specially appointed independent committee for consideration, comment and guidance.

3. Biomedical research involving human subjects should be conducted only by scientifically qualified persons and under the supervision of a clinically competent medical person. The responsibility for the human subject must always rest with a medically qualified person and never rest on the subject of the research, even though the subject has given his or her consent.

4. Biomedical research involving human subjects cannot legitimately be carried out unless the importance of the objective is in proportion to the inherent risk to the subject.

5. Every biomedical research project involving human subjects should be preceded by careful assessment of predictable risks in comparison with foreseeable benefits to the subject or to others. Concern for the interests of the subject must always prevail over the interests of science and society.

6. The right of the research subject to safeguard his or her integrity must always be respected. Every precaution should be taken to respect the privacy of the subject and to minimize the impact of the study on the subject's physical and mental integrity and on the personality of the subject.

7. Physicians should abstain from engaging in research projects involving human subjects unless they are satisfied that the hazards involved are believed to be predictable. Physicians should cease any investigation if the hazards are found to outweigh the potential benefits.

8. In publication of the results of his or her research, the physician is obliged to preserve the accuracy of the results. Reports of experimentation not in accordance with the principles laid down in this Declaration should not be accepted for publication.

9. In any research on human beings, each potential subject must be adequately informed of the aims, methods, anticipated benefits and potential hazards of the study and the discomfort it may entail. He or she should be informed that he or she is at liberty to abstain from participation in the study and that he or she is free to withdraw visor her consent to participation at any time. The physician should then obtain the subject's freely given informed consent, preferably inheriting.

10. When obtaining informed consent for the research project the physician should be particularly cautious if the subject is in dependent relationship to him or her or may consent under duress. In that case the informed consent should be obtained by a physician who isn't engaged in the investigation and who is completely independent of this official relationship.

11. In case of legal incompetence, informed consent should be obtained from the legal guardian in accordance with national legislation. Where physical or mental incapacity makes it impossible to obtain informed consent, or when the subject is a minor, permission from the responsible relative replaces that of the subject in accordance with national legislation. Whenever the minor child is in fact able to give consent, the minor's consent must be obtained in addition to the consent of the minor's legal guardian.

12. The research protocol should always contain a statement of the ethical considerations involved and should indicate that the principles enunciated in the present declaration are complied with.

II. Medical Research Combined with Professional Care (Clinical Research)

1. In the treatment of the sick person, the physician must be free to use a new diagnostic and therapeutic measure, if in his or her judgement it offers hope of saving life, re-establishing health or alleviating suffering.

2. The potential benefits, hazards and discomfort of a new method should be weighed against the advantages of the best current diagnostic and therapeutic methods.

3. In any medical study, every patient- including those of a control group, if any- should be assured of the best proven diagnostic and therapeutic method.

4. The refusal of the patient to participate in a study must never interfere with the physician-patient relationship.

5. If the physician considers it essential not to obtain informed consent, the specific reasons for this proposal should be stated in the experimental protocol for transmission to the independent committee (1, 2).

6. The physician can combine medical research with professional care, the objective being the acquisition of new medical knowledge, only to the extent that medical research is justified by its potential diagnostic or therapeutic value for the patient.

III. Non-Therapeutic Biomedical Research Involving Human Subjects (Non-Clinical Biomedical Research)

1. In the purely scientific application of medical research carried out on a human being, it is the duty of the physician to remain the protector of the life and health of that person on whom biomedical research is being carried out.

2. The subjects should be volunteers- either healthy persons or patients for whom the experimental design is not related to the patient's illness.

3. The investigator or the investigating team should discontinue the research if in his/her or their judgment it may, if continued, be harmful to the individual.

4. In research on man, the interest of science and society should never take precedence over considerations related to the well-being of the subject.

Informed Consent

The Investigator will obtain informed consent from each subject enrolled in the study, in accordance with the Declaration of Helsinki, the current versions of the ICH guidelines and the laws and regulations of the country in which the investigation is being conducted.

The Ethics Committee must accept the informed consent document to be used by the Investigator. It is the responsibility of the Investigators to assure that the patient (or guardian or legal representative) has signed the Informed Consent before any activity or treatment is undertaken which is not part of routine care. This includes, but is not limited to, the performance of diagnostic ort therapeutic procedures and the administration of the first dose of the study medication.

Appendix 6 Essential Documents

Prior to the beginning of any clinical study, the Investigator will be asked to provide the following documents to STI:

A current *curriculum vita* of the investigator. If sub-investigators will participate in the study, curriculum *vitae* for each are to be provided.

Requirements for the Independent Ethics Committee (IEC).

A copy of the signed and dated letter of approval of the IEC. The letter must specify that both the protocol and informed consent form were approved (unless separate documentation that the informed consent was approved is provided).

A dated list containing the names and affiliations of the members of the IEC.

11. References

1. WHO. The director-general of WHO calls for faster production of drug to treat sleeping sickness. *WHO Press* 1996;**25**(March 27, 1996).

2. WHO. Control and surveillance of African trypanosomiasis. Geneva: WHO, 1998.

3. Bales JD, Jr. African Trypanosomiasis. In: G.T. S, ed. Hunter's Tropical Medicine. 7 ed. Philadelphia: Saunders Company, 1988: 617-628.

4. Molyneux DH, Pentreath V, Doua F. African trypanosomiasis in man. In: Cook GC, ed. Manson's Tropical Diseases. 20 ed. London: W.B. Saunders Company Ltd., 1996: 1171-1196.

5. Braendli B, Dankwa E, Junghans T. Ostafrikanische Schlafkrankheit (Infektion mit Trypansoma rhodesiense) bei zwei schweizerischen Tropenreisenden. *Schweiz. Med. Wschr.* 1990;**120:**1348-1352.

6. Wery M. Therapy for African trypanosomiasis. *Current Opinion in Infectious Diseases* 1991;**4**(6)**:**838-844.

7. Chappuis F, Loutan L, Simarro P, Lejon V, Buscher P. Options for field diagnosis of human african trypanosomiasis. *Clin Microbiol Rev* 2005;**18**(1)**:**133-46.

8. Burri C, Stich A, Brun R. The trypanosomiases: CABI Publishing, 2004.

9. Odiit M, Kansiime F, Enyaru JC. Duration of symptoms and case fatality of sleeping sickness caused by Trypanosoma brucei rhodesiense in Tororo, Uganda. *East Afr Med J* 1997;**74**(12)**:**792-5.

10. Greenwood BM, Whittle HC. The pathogenesis of sleeping sickness. *Trans R Soc Trop Med Hyg* 1980;**74**(6)**:**716-25.

11. Chijioke CP, Umeh RE, Mbah AU, Nwonu P, Fleckenstein LL, Okonkwo PO. Clinical pharmacokinetics of suramin in patients with onchocerciasis. *Eur J Clin Pharmacol* 1998;**54**(3)**:**249-51.

12. Nightingale S. Drug for sleeping sickness approved. *Journal of the American Medical Association* 1991;**265**(10)**:**1229.

13. Iten M, Matovu E, Brun R, Kaminsky R. Innate lack of susceptibility of Ugandan Trypanosoma brucei rhodesiense to DL-alpha-difluoromethylornithine (DFMO). *Trop Med Parasitol* 1995;**46**(3)**:**190-4.

14. Seixas J. Investigations on the encephalopathic syndrome during melarsoprol treatment in human african trypanosomiasis: Base, Lisabon, 2004.

15. Pepin J, Milord F. African trypanosomiasis and drug-induced encephalopathy: risk factors and pathogenesis. *Trans R Soc Trop Med Hyg* 1991;**85**(2)**:**222-4.

16. WHO T. http://www.who.int/tdr/diseases/tryp/direction.htm, 2002.

17. Burri C, Baltz T, Giroud C, Doua F, Welker HA, Brun R. Pharmacokinetic properties of the trypanocidal drug melarsoprol. *Chemotherapy* 1993;**39**(4)**:**225-234.

18. Keiser J, Ericsson O, Burri C. Investigations of the metabolites of the trypanocidal drug melarsoprol. *Clinical Pharmacology and Therapeutics* 2000;**67**(5)**:**478-88.

19. Keiser J, Stich A, Burri C. New drugs for the treatment of human African trypanosomiasis: research and development. *Trends Parasitol* 2001;**17**(1)**:**42-9.

20. Burri C. Pharmacological aspects of the trypanocidal drug melarsoprol: University of Basel, 1994.

21. Schmid C, Nkunku S, Merolle A, Vounatsou P, Burri C. Efficacy of 10-day melarsoprol schedule 2 years after treatment for late-stage gambiense sleeping sickness. *Lancet* 2004;**364**(9436)**:**789-90.

22. Fevre EM. Strengthening Health Systems by Cooperation between Human and Animal Health. Basel

2005.

23. Odiit M, Coleman PG, Liu WC, et al. Quantifying the level of under-detection of Trypanosoma brucei rhodesiense sleeping sickness cases. *Trop Med Int Health* 2005;**10**(9)**:**840-9.

24. WHO. Human African trypanosomiasis (sleeping sickness): epidemiological update, 2006: 69-80.

25. Picozzi K, Fevre EM, Odiit M, et al. Sleeping sickness in Uganda: a thin line between two fatal diseases. *Bmj* 2005;**331**(7527)**:**1238-41.

26. Fevre EM, Coleman PG, Odiit M, Magona JW, Welburn SC, Woolhouse ME. The origins of a new Trypanosoma brucei rhodesiense sleeping sickness outbreak in eastern Uganda. *Lancet* 2001;**358**(9282)**:**625-8.

27. Legros D, Gastellu Etchegorry M, Mbulamberi DB. Preliminary results of a clinical trial comparing melarsoprol to pentamidine for the treatment of early stage two *T. b. gambiense* patients in Uganda. International Colloquium "Sleeping Sickness Rediscovered" 1998, Antwerp.

28. Kibona SN, Matemba L, Kaboya JS, Lubega GW. Drug-resistance of Trypanosoma b. rhodesiense isolates from Tanzania. *Trop Med Int Health* 2006;**11**(2)**:**144-55.

29. Jelinek T, Bisoffi Z, Bonazzi L, et al. Cluster of African trypanosomiasis in travelers to Tanzanian national parks. *Emerg Infect Dis* 2002;**8**(6)**:**634-5.

30. Schmid C, Santercole C, Kwete J, Lutumba P, Shaw AP. An economic appraisal of the late-stage *T.b. gambiense* sleeping sickness treatment. *in preparation* 2005.

31. Pentreath VW, Owolabi AO, Doua F. Survival of Trypanosoma brucei brucei in cerebrospinal fluid. *Ann Trop Med Parasitol* 1992;**86**(1)**:**29-34.

32. Pepin J, Ethier L, Kazadi C, Milord F, Ryder R. The impact of human immunodeficiency virus infection on the epidemiology and treatment of Trypanosoma brucei gambiense sleeping sickness in Nioki, Zaire. *Am J Trop Med Hyg* 1992;**47**(2)**:**133-40.

33. Jäggi M. Zeit/Dosis Abhängigkeit der Wirkung trypanozider Medikamente auf afrikanische Trypanosomen. *Diploma Thesis, Swiss Tropical Institute, Basel* 1991.

34. Schmid C, Richer M, Bilenge CM, et al. Effectiveness of a 10-Day Melarsoprol Schedule for the Treatment of Late-Stage Human African Trypanosomiasis: Confirmation from a Multinational Study (Impamel II). *J Infect Dis* 2005;**191**(11)**:**1922-31.

35. Matovu E, Enyaru JC, Legros D, Schmid C, Seebeck T, Kaminsky R. Melarsoprol refractory T. b. gambiense from Omugo, north-western Uganda. *Trop Med Int Health* 2001;**6**(5)**:**407-11.

36. Welburn SC, Picozzi K, Fevre EM, et al. Identification of human-infective trypanosomes in animal reservoir of sleeping sickness in Uganda by means of serum-resistance-associated (SRA) gene. *Lancet* 2001;**358**(9298)**:**2017-9.

37. Chappuis F, Udayraj N, Stietenroth K, Meussen A, Bovier PA. Eflornithine is safer than melarsoprol for the treatment of second-stage Trypanosoma brucei gambiense human African trypanosomiasis. *Clin Infect Dis* 2005;**41**(5)**:**748-51.

38. Woo PT. The haematocrit centrifuge for the detection of trypanosomes in blood. *Canadian Journal of Zoology* 1969;**47**(5)**:**921-3.

39. Ancelle T, Barret B, Flachet L, Moren A. [2 epidemics of arsenical encephalopathy in the treatment of trypanosomiasis, Uganda, 1992-1993]. *Bull Soc Pathol Exot* 1994;**87**(5)**:**341-6.

40. WHO. Epidemiology and control of African trypanosomiasis. *WHO Technical Report Series* 1986;**739**.

41. Miezan TW, Meda HA, Doua F, Dje NN, Lejon V, Buscher P. Single centrifugation of cerebrospinal fluid in a sealed pasteur pipette for simple, rapid and sensitive detection of trypanosomes. *Trans R Soc Trop Med Hyg* 2000;**94**(3)**:**293.

42. Horton R, Smith R. Signing up for authorship. *Lancet* 1996;**347:**780.
